# Phylogeny and biogeography of a shallow water fish clade (Teleostei: Blenniiformes)

**DOI:** 10.1186/1471-2148-13-210

**Published:** 2013-09-25

**Authors:** Hsiu-Chin Lin, Philip A Hastings

**Affiliations:** 1 Marine Biology Research Division, Scripps Institution of Oceanography, University of California San Diego, La Jolla, CA 92093, USA; 2 Biodiversity Research Center, Academia Sinica, Taipei 115, Taiwan

**Keywords:** Tripterygiidae, Blenniidae, Labrisomidae, Clinidae, Dactyloscopidae, Chaenopsidae, Neotropics, Tethys sea, Biogeography

## Abstract

**Background:**

The Blenniiformes comprises six families, 151 genera and nearly 900 species of small teleost fishes closely associated with coastal benthic habitats. They provide an unparalleled opportunity for studying marine biogeography because they include the globally distributed families Tripterygiidae (triplefin blennies) and Blenniidae (combtooth blennies), the temperate Clinidae (kelp blennies), and three largely Neotropical families (Labrisomidae, Chaenopsidae, and Dactyloscopidae). However, interpretation of these distributional patterns has been hindered by largely unresolved inter-familial relationships and the lack of evidence of monophyly of the Labrisomidae.

**Results:**

We explored the phylogenetic relationships of the Blenniiformes based on one mitochondrial (COI) and four nuclear (TMO-4C4, RAG1, Rhodopsin, and Histone H3) loci for 150 blenniiform species, and representative outgroups (Gobiesocidae, Opistognathidae and Grammatidae). According to the consensus of Bayesian Inference, Maximum Likelihood, and Maximum Parsimony analyses, the monophyly of the Blenniiformes and the Tripterygiidae, Blenniidae, Clinidae, and Dactyloscopidae is supported. The Tripterygiidae is the sister group of all other blennies, and the Blenniidae is the sister group of the remaining blennies. The monophyly of the Labrisomidae is supported with the exclusion of the Cryptotremini and inclusion of *Stathmonotus,* and we elevate two subgenera of *Labrisomus* to establish a monophyletic classification within the family. The monophyly of the Chaenopsidae is supported with the exclusion of *Stathmonotus* (placed in the Stathmonotini) and *Neoclinus* and *Mccoskerichthys* (placed in the Neoclinini). The origin of the Blenniiformes was estimated in the present-day IndoPacific region, corresponding to the Tethys Sea approximately 60.3 mya. A largely Neotropical lineage including the Labrisomidae, Chaenopsidae and Dactyloscopidae (node IV) evolved around 37.6 mya when the Neotropics were increasingly separated from the IndoPacific, but well before the closure of the Tethys Sea.

**Conclusions:**

Relationships recovered in this study are similar to those of earlier analyses within the Clinidae and Chaenopsidae, and partially similar within the Blenniidae, but tripterygiid relationships remain poorly resolved. We present the first comprehensive phylogenetic hypothesis for a monophyletic Labrisomidae with five tribes (Labrisomini, Mnierpini, Paraclinini, Stathmonotini and Starksiini). Global distributions of blenny genera included in our analysis support the evolution of a largely Neotropical clade whose closest relatives (clinids and cryptotremines) are temperate in distribution.

## Background

The Blenniiformes comprises six families, 151 genera and nearly 900 species [1] of small teleost fishes closely associated with coastal benthic habitats. Although small, blennies often numerically dominate rocky reef ichthyofaunas [2-4]. Because of their abundance and the ease at which they can be observed, blennies have become convenient models for the study of ecology and behavior [5]. Blennies also provide an unparalleled opportunity for marine biogeography because they include the globally distributed families Tripterygiidae and Blenniidae, the temperate Clinidae, and three families (Labrisomidae, Chaenopsidae, and Dactyloscopidae) largely restricted to the Neotropics [6]. The breakup of the Tethys Sea [7], formation of the Central America Isthmus [8], and climate warming during the Pliocene [9] have been suggested as important historical events shaping the evolution and current distributional patterns of blennies. However, insights from these and other studies have been compromised by the absence of a well-resolved phylogeny for the group.

The history of blenniiform classification has been reviewed by several researchers (e.g. [1,10-12]). Members of the currently recognized families Tripterygiidae, Blenniidae, Labrisomidae, Clinidae and Chaenopsidae are consistently included, but other families have been added depending on the definition of “true” blennies [11,13-16]. A widely accepted concept of a monophyletic Blenniiformes as the “tropical” blenny families (= Blenniicae *sensu* Hubbs, 1952; [14]) was revisited by Springer [11] and, based on morphological characters, formalized to include the above five families and the sand stargazers, Dactyloscopidae, as hypothesized earlier by Regan [16]. Springer’s Blennioidei, termed the Blenniiformes by Wiley and Johnson [17], shares several unique morphological features including presence of a bean-shaped pelvis, a reduced branchial apparatus, proximal pectoral-fin radials longer than wide, unbranched pectoral-fin rays, relatively simple caudal-fin morphology, 0–2 spines and simple segmented rays in the anal-fin, and no neural spine on the first vertebra [11,18].

Inter-familial relationships of blenniiforms have remained largely unresolved because of conflicting morphological and molecular evidence [1]. It is generally agreed that the Tripterygiidae is the sister group of all other blennies, based on these groups having unbranched dorsal- and pectoral-fin rays (branched in most triplfins), no ctenoid scales as in triplefins, and roofed sensory canal bones (unroofed in triplefins) [11]. It has also been hypothesized that the Blenniidae is the sister group of the remaining blennies based on several features of the dorsal gill-arches and associated muscles [19]. However, a consensus has not been reached regarding relationships among the Clinidae, Chaenopsidae, Dactyloscopidae and Labrisomidae. The monophyly of the first three of these families has been supported by morphological synapomorphies [11]. However, the Labrisomidae includes generalized blennies that do not fall into the other relatively well-defined families and no synapomorphies have been identified for this group [11] other than one possible reductive character [20]. Labrisomids have long been considered to be closely related to the Clinidae (e.g. [14]) and included in that family by some authors (e.g. [21]). In addition, the relationships of the Dactyloscopidae to other blennies, the most recent major lineage to be added to the Blenniiformes [11], have been evaluated based only on dorsal gill-arch anatomy [19]. Thus, dactyloscopids have been placed in an unresolved polytomy along with the Clinidae, Labrisomidae and Chaenopsidae [1]. Two molecular studies have attempted to resolve the phylogenetic relationships among the blenny families [9,22]. These provided inconsistent phylogenetic hypotheses, although both questioned the monophyly of the Labrisomidae and the Chaenopsidae.

Recently, the systematics of blennies was thoroughly reviewed, providing convenient reference points: the Labrisomidae, Clinidae, Chaenopsidae, and Dactyloscopidae by Hastings and Springer [1], the Blenniidae by Hastings and Springer [23] and the Tripterygiidae by Fricke [24]. In addition, studies on higher-level relationships of fishes based on morphology and multiple genetic markers have suggested a close relationship between blennies and clingfishes (Gobiesocidae) [19,25-30], jawfishes (Opistognathidae) [31,32], and basslets (Grammatidae) [31,33-35] within the recently recognized Ovalentaria [32]. In the present study, we attempt to reconstruct the phylogenetic relationships of the Blenniiformes with significantly broader taxon sampling (150 blenniiform species), substantially more genetic information (one mitochondrial and four nuclear markers), and more strategic outgroup representation than in previous studies.

## Results

### Sequence analysis

The dataset comprises 150 blenniiforms, six gobiesocids, one opistognathid, and one grammatid species as terminal taxa scored for 3,562 bp including 570 bp in COI, 421 bp in TMO-4C4, 1,506 bp in RAG1, 737 bp in Rhodopsin, and 328 bp in Histone H3 (Table 1 and Additional file 1: Table S1). Genetic markers unable to be amplied and sequenced (14% in COI, 15% in TMO-4C4, 6% in RAG1 and Rhodopsin, and 4% in Histone H3) were treated as missing data for all phylogenetic analyses. The alignments of COI, Rhodopsin and Histone H3 were unambiguous, but several indels with multiples of three were observed in TMO-4C4 and RAG1, especially in species of the Tripterygiidae. The aligned data matrix is available in TreeBASE (http://purl.org/phylo/treebase/phylows/study/TB2:S14731). The GC content of the first codon across all markers ranged from 51.1% to 61.9%, the second codon from 32.4% to 47.7%, and the third codon from 39.2% to 84.0% (Table 2). The Chi-square test results of base-frequency homogeneity across taxa were significant for the third codon position of COI, TMO-4C4, Rhodopsin and RAG1 (p < 0.005), but not significant for the remaining partitions.

**Table 1:** **Currently recognized lineages of six families of the Blenniiformes **[1]**,**[23]**,**[24]**,**[36]

**Family (total genera/ total species)**	**Lineage (total genera/ sampled genera)**	**Genera (total species/ sampled species)**
Tripterygiidae (32/164)		
	Notoclinini (2/0)	
	Trianectini (5/0)	
	Norfolkiini (4/2)	*Cremnochorites* (1/1)
		*Lepidonectes* (3/1)
	Tripterygiini (8/4)	*Axoclinus* (6/3)
		*Crocodilichthys* (1/1)
		*Enneanectes* (8/7)
		*Enneapterygius* (53/3)
	Forsterygiini (5/0)	
	Karalepini (2/0)	
	Helcogrammini (3/1)	*Helcogramma* (39/2)
	Blennodontini (3/0)	
		
Blenniidae (57/387)		
	Blenniini (2/0)	
	Nemophini (5/4)	*Meiacanthus* (25/1)
		*Petroscrites* (11/1)
		*Plagiotremus* (11/3)
		*Xiphasia* (2/1)
	Omobranchini (7/1)	*Omobranchus* (21/2)
	Phenablenniini (1/0)	
	Parablenniini (14/2)	*Hypsoblennius* (14/6)
		*Parablennius* (27/1)
	Salariini-Salarias group (20/12)	*Alticus* (10/1)
		*Andamia* (7/2)
		*Atrosalarias* (3/1)
		*Blenniella* (9/2)
		*Cirripectes* (22/5)
		*Ecsenius* (53/4)
		*Entomacrodus* (25/4)
		*Istiblennius* (14/3)
		*Nannosalarias* (1/1)
		*Ophioblennius* (5/2)
		*Praealticus* (13/5)
		*Salarias* (13/2)
	Salariini-Rhabdoblennius group (8/1)	*Rhabdoblennius* (5/1)
Labrisomidae (14/109)		
	Cryptotremini (4/3)	*Alloclinus* (1/1)
		*Auchenionchus* (3/1)
		*Calliclinus* (2/1)
	Labrisomini (2/2)	*Labrisomus* (20/7)
		*Malacoctenus* (21/10)
	Paraclinini (2/2)	*Exerpes* (1/1)
		*Paraclinus* (23/4)
	Starksiini (2/2)	*Starksia* (30/9)
		*Xenomedea* (1/1)
	Mnierpini (2/1)	*Dialommus* (2/1)
	Uncertain (2/0)	
Clinidae (26/85)		
	Clinini (17/7)	*Blennioclinus* (2/1)
		*Blennophis* (2/1)
		*Clinus* (17/2)
		*Cristiceps* (3/1)
		*Heteroclinus* (15/2)
		*Muraenoclinus* (1/1)
		*Pavoclinus* (9/2)
	Myxodini (5/2)	*Gibbonsia* (3/3)
		*Heterostichus* (1/1)
	Ophiclinini (4/0)	
Chaenopsidae (14/91)		
	Chaenopsinae (11/10)	*Acanthemblemaria* (20/6)
		*Chaenopsis* (10/2)
		*Cirriemblemaria* (1/1)
		*Coralliozetus* (6/6)
		*Ekemblemaria* (3/2)
		*Emblemaria* (16/3)
		*Emblemariopsis* (12/2)
		*Hemiemblemaria* (1/1)
		*Lucayablennius* (1/1)
		*Protemblemaria* (3/1)
	Uncertain (3/3)	*Mccoskerichthys* (1/1)
		*Neoclinus* (9/1)
		*Stathmonotus* (7/3)
Dactyloscopidae (9/48)		
	Dactyloids (3/1)	*Dactyloscopus* (20/2)
	Gillelloids (3/1)	*Gillellus* (10/1)
	Uncertain (3/1)	*Platygillellus* (6/1)

Only the genera with representative taxa included in this study are listed.

**Table 2:** Proportions of variable and parsimony informative characters, best nucleotide substitution model and parameters selected by jModelTest for each data partition

**Gene**	**Codon position**	**Length (bp)**	**G+C (%)**	**Variable characters**	**Parsimony informative characters**	**Best model by jModelTest**	**I**	**G**
COI	1st+2nd	380	55.2/43.2	74 (19.47%)	51 (13.42%)	TIM3+I+G	0.7510	0.4720
	3rd	190	39.2	190 (100%)	189 (99.47%)	GTR+G	-	0.3310
TMO-4C4	1st+2nd	280	59.0/32.4	114 (40.71%)	79 (28.21%)	TIM3+I+G	0.4190	0.4040
	3rd	140	63.3	137 (97.86%)	135 (96.32%)	TVM+I+G	0.0210	1.2690
Rhod	1st+2nd	492	51.1/42.4	66 (13.41%)	55 (11.18%)	TIM3+I+G	0.8340	0.6190
	3rd	245	76.9	176 (71.84%)	154 (62.86%)	TVM+I+G	0.2050	0.6940
Rag1	1st+2nd	1004	56.2/41.2	307 (30.58%)	223 (22.21%)	GTR+I+G	0.5550	0.3760
	3rd	502	66.9	455 (90.64%)	432 (86.06%)	TPM2uf+I+G	0.0810	0.9380
H3	1st+2nd	218	61.9/47.7	21 (9.63%)	9 (4.13%)	GTR+I+G	0.7270	0.1840
	3rd	110	84.0	76 (69.09%)	65 (59.09%)	TIM2+I+G	0.2570	0.7580

I: proportion of invariant sites. G: gamma rate variation among sites.

Among the 3,562 bp, the alignment comprised 1,627 (45.68%) variable sites, of which 1,392 (39.08%) were parsimony informative. The sequences of the ten partitions provided a range of evolutionary information. The third codon position of all five markers provided significantly more parsimony information (59.09% to 99.47%) than the first and second positions (4.13% to 28.21%). The third codon of the only mitochondrial marker (COI) had the highest proportion of variable characters (100%) and the highest proportion of parsimony informative characters (99.47%). The first and second codon positions of the nuclear marker Histone H3 had the lowest proportion of both variable characters (9.63%) and parsimony informative characters (4.13%).

### Phylogenetic relationships

**Figure 1:**
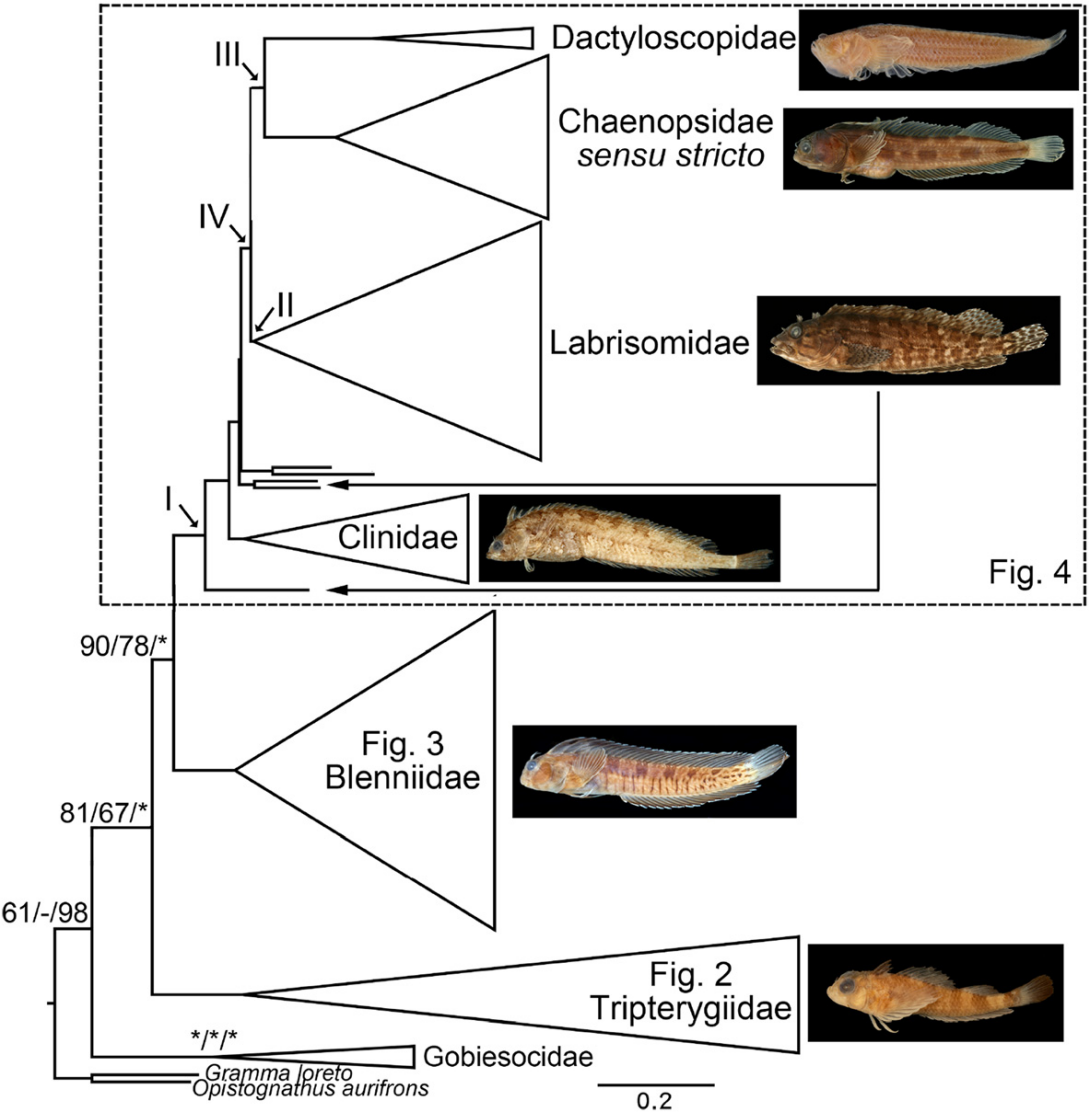
**Phylogenetic relationships among major blenniiform clades based on Maximum Likelihood analysis.** The supporting values of nodes (Maximum Likelihood bootstrap/Maximum Parsimony bootstrap/Bayesian posterior probability) were only shown if not in Figures 2, 3 and 4 where the phylogenetic relationships are shown in detail. *=100. - = not supported. Representative photos of the six blenniiform families are *Enneanectes macrops* (Tripterygiidae), *Hypsoblennius striatus* (Blenniidae), *Myxodes ornatus* (Clinidae), *Labrisomus xanti* (Labrisomidae), *Acanthemblemaria hastingsi* (Chaenopsidae), and *Dactyloscopus lunaticus* (Dactyloscopidae).

**Figure 2:**
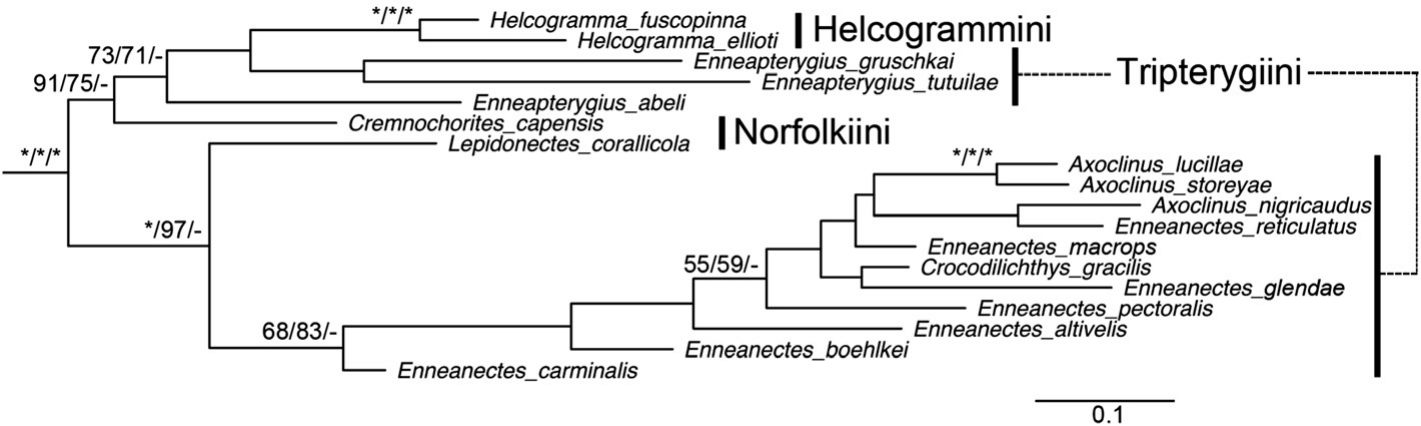
**Phylogenetic relationships of the Tripterygiidae based on Maximum Likelihood analysis.** Node supports are Maximum Likelihood bootstrap/Maximum Parsimony bootstrap/Bayesian posterior probability. *=100. - = not supported.

**Figure 3:**
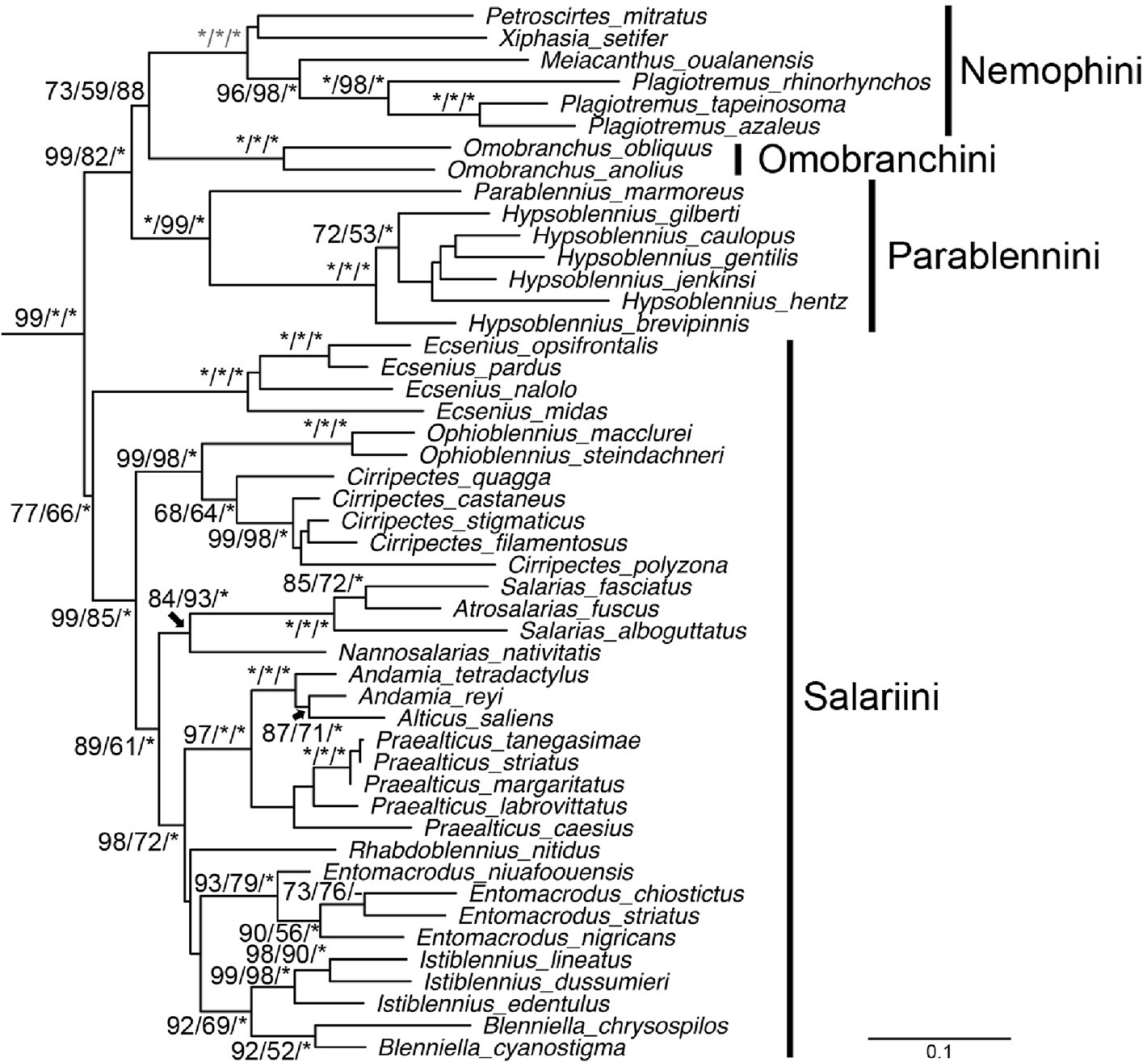
**Phylogenetic relationships of the Blenniidae based on Maximum Likelihood analysis.** Node supports are Maximum Likelihood bootstrap/Maximum Parsimony bootstrap/Bayesian posterior probability. *=100. - = not supported.

**Figure 4:**
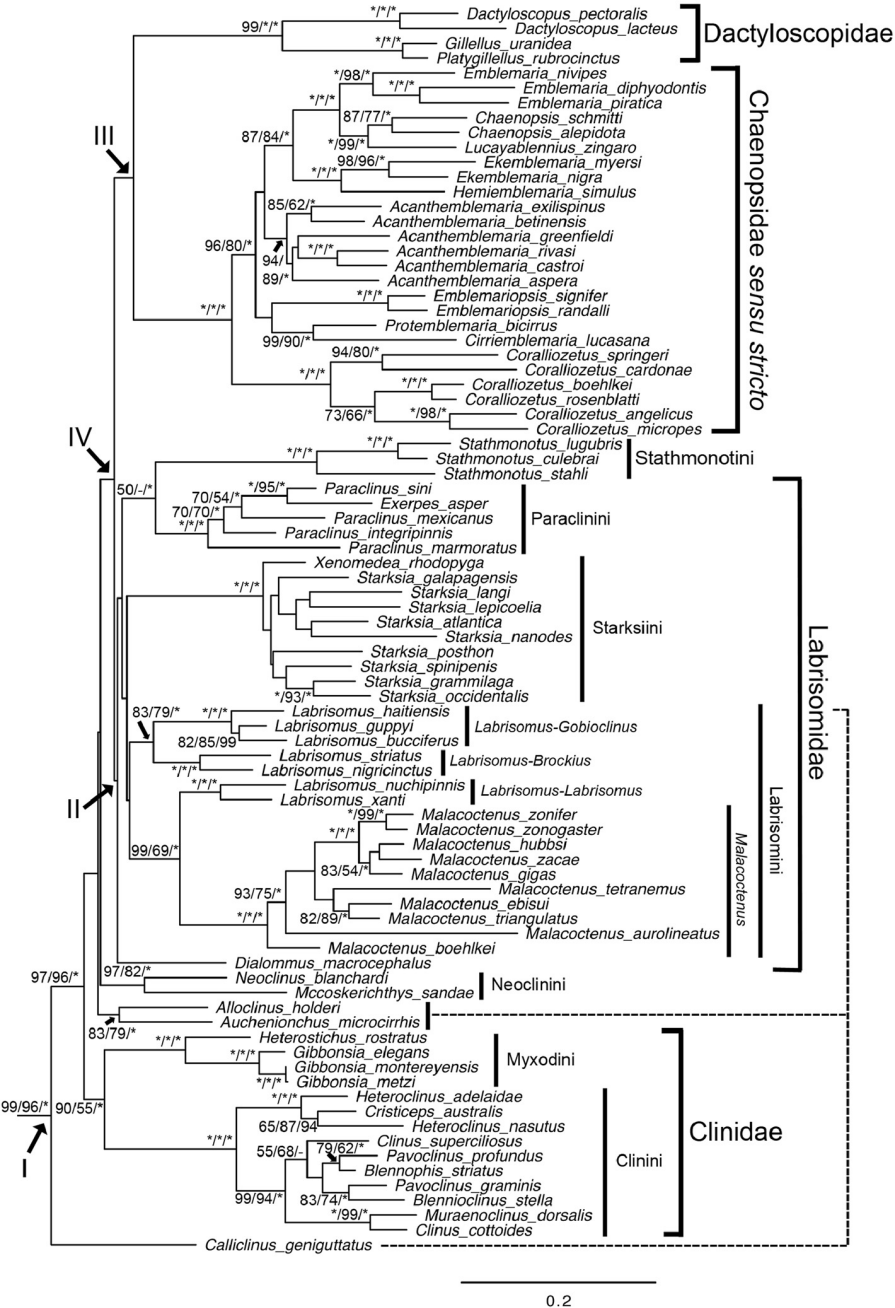
**Phylogenetic relationships of the blenniiform species other than Tripterygiidae and Blenniidae based on Maximum Likelihood analysis.** Node supports are Maximum Likelihood bootstrap/Maximum Parsimony bootstrap/Bayesian posterior probability. *=100. - = not supported.

For the complete dataset, the score of the best ML trees found was −80231.7055. Four equally parsimonious trees of 18,493 steps were returned with the MP analysis. Both ML- and MP-generated topologies based on the complete dataset are congruent with those from BI analysis with regard to the relationships at the family-, subfamily-, and tribe-levels. The recovered relationships of blenniiform clades based on maximum likelihood methods are summarized in Figure 1 and in detail in Figures 2, 3 and 4. The concatenated molecular data strongly support the monophylies of the Tripterygiidae (ML bootstrap value / MP bootstrap value / Bayesian posterior probability = 100/100/100), Blenniidae (99/100/100), Clinidae (90/55/100), Chaenopsidae *sensu stricto* (100/100/100), and Dacty-loscopidae (99/100/100), as well as the monophyly of their sister group, the Gobiesocidae (100/100/100). The Tripterygiidae is the sister group of all other blenniiforms, and the Blenniidae is the sister group of all remaining blennies. *Calliclinus geniguttatus* (currently a labrisomid) is the sister group of a large clade that includes the Clinidae, Labrisomidae, Chaenopsidae and Dactyloscopidae (Figure 4).

In general, the relationships among triplefins were poorly resolved in all analyses (Figure 2). Relationships within the Blenniidae were, however, well resolved, with only a few discrepancies among analytical methods (Figure 3). The monophyly of all blenniid genera for which we sampled multiple species was well supported except that *Alticus saliens* was nested within *Andamia,* and *Atrosalaris fuscus* was nested within *Salarias* (Figure 3). The monophyly of each the four blenniid tribes included in our study (i.e., Parablenniini, Salariini, Nemophini, and Omobranchini) was well supported with the exception of the Salariini in the MP analysis (MP bootstrap value = 66). The two Blenniinae tribes Nemophini and Omobranchini were grouped together and were the sister group of the tribe Parablenniini. This entire clade was the sister group of the tribe Salariini.

Relationships among the remaining four families were well resolved in the shallower nodes, but less so in the deeper nodes (Figure 4). In general the pattern is the inclusion of low-diversity taxa as the sister group of larger monophyletic clades. These low-diversity groups include *Calliclinus* (Cryptotremini), *Auchenionchus* + *Alloclinus* (both currently Cryptotremini), and the “chaenopsids” *Neoclinus* and *Mccoskerichthys*. In addition to a monophyletic Clinidae, all three analytical methods recovered two diverse clades with moderate support values. These included the members of the Labrisomidae (node II) other than the Cryptotremini that together formed a monophyletic group with the inclusion of the genus *Stathmonotus* (currently considered a chaenopsid). The second lineage (node III) included the Dactyloscopidae plus the Chaenopsidae *sensu stricto* (i.e., excluding *Stathmonotus*, *Neoclinus* and *Mccoskerichthys*).

Within the labrisomid clade (node II; BI PP=74), BI recovered relationships with *Dialommus* (Mnierpini) as the sister group of two relatively large clades, the Paraclinini + *Stathmonotus*, and Starksiini + Labrisomini (Figure 4). Within the Paraclinini, the monotypic genus *Exerpes* was nested within the genus *Paraclinus*, while within the Starksiini, the monotypic genus *Xenomedea* was the sister group of a monophyletic *Starksia* (Figure 4). Within the Labrisomini, the genus *Labrisomus* was not monophyletic, but divided into two clades. One clade included the subgenus *Labrisomus* that was the sister group of the monophyletic genus *Malacoctenus*, while the other clade included all other sampled members of *Labrisomus* (Figure 4).

In the remaining large clade (node III; BI PP=86), the monophyletic Dactyloscopidae was the sister group of a monophyletic Chaenopsidae *sensu stricto* (i.e., exclusive of *Stathmonotus*, *Neoclinus* and *Mccoskerichthys*). Within the Chaenopsidae all currently recognized genera were recovered as monophyletic, and the genus *Coralliozetus* was the sister group of all other chaenopsids. That group included two large clades, one with *Emblemariopsis*, *Protemblemaria* and *Cirriemblemaria*, and another with *Acanthemblemaria*, *Ekemblemaria*, *Hemiemblemaria*, *Emblemaria*, *Chaenopsis* and *Lucayablennius* (Figure 4).

### Biogeography

The divergence times of blenniiform lineages were estimated from the combined postburn-in trees and parameter values of the BEAST analyses (Additional file 2: Figure S1). The maximum clade credibility tree estimated from the posterior density was characterized by a large number of nodes supported with significant Bayesian posterior probabilities (data not shown) and the mean of the posterior density of the likelihood score was −78678.5184 (95% highest posterior densities [HPD]: -78748.29 to −78607.9508).

**Figure 5:**
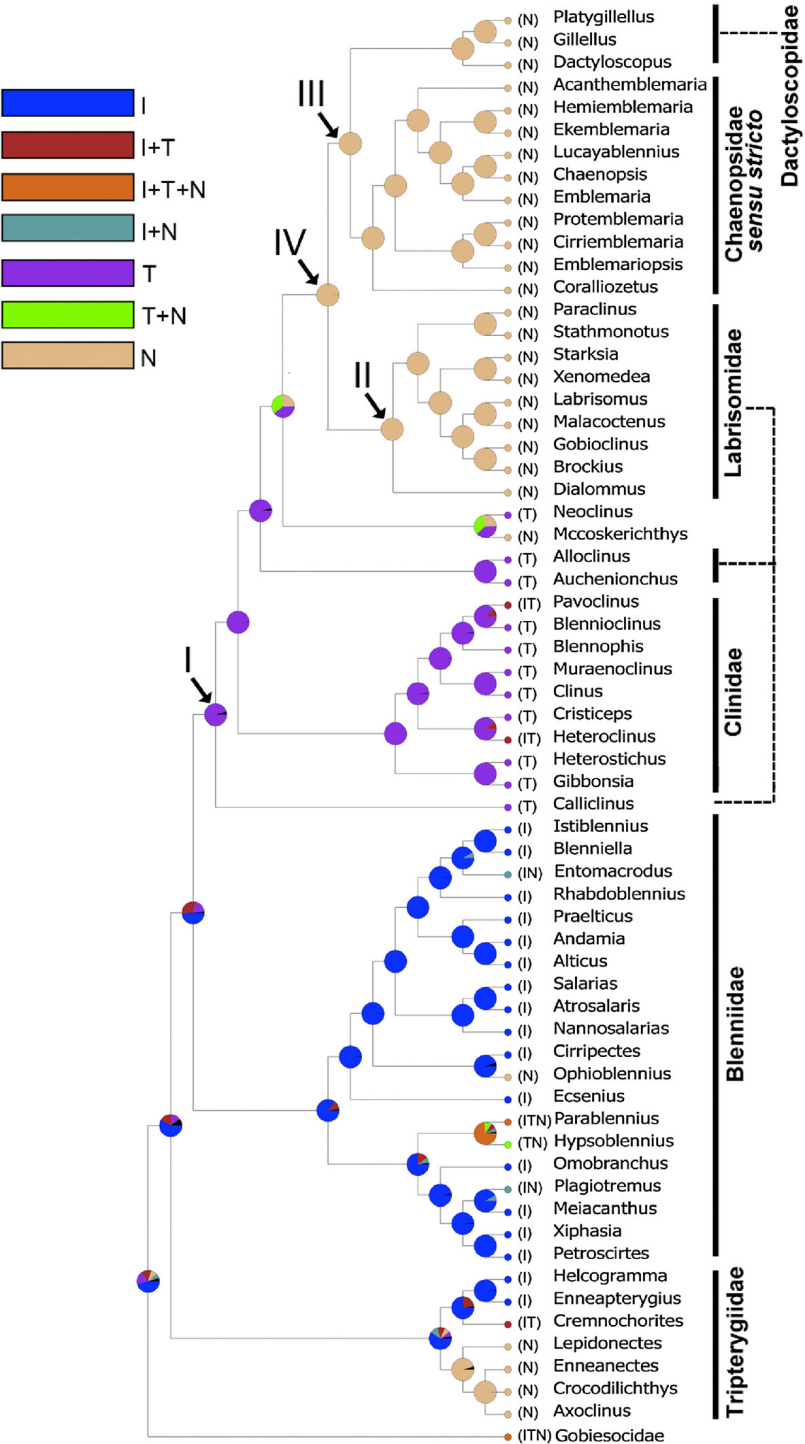
**Inferred ancestral distribution at each node of the blenniiform phylogeny estimated by BBM analysis implemented in RASP.** Pie charts at each node indicate probabilities of alternative ancestral distributions. I: tropical IndoPacific, T: temperate, N: Neotropics.

Mapping present day distributions of genera included in our study using BBM (Figure 5) and MP (Additional file 3: Figure S2) indicate: 1) an IndoPacific origin for the Blenniiformes with an estimated age of 60.3 my (HPD: 28.2 to 95.9); 2) multiple invasions of the Neotropics and/or temperate regions within the Tripterygiidae and Blenniidae that occurred at a variety of times before the present; 3) a Neotropical origin of the clade including the Labrisomidae, Chaenopsidae and Dactyloscopidae (node IV) with an estimated age 37.6 my (HPD: 17.5 to 59.7); 4) primarily temperate origins for the intervening clades including the Clinidae and current members of the nonmonophyletic Cryptotremini.

## Discussion

### Families and inter-family relationships of the Blenniiformes

Although the monophyly of the Blenniiformes *sensu* Wiley and Johnson, 2009 [17] (= Blennioidei *sensu* Springer, 1993; [11]) has been questioned by some (e.g. [34]), our phylogenetic analysis found strong support for its monophyly in agreement with other recent molecular analyses (e.g. [32,35]). Blenniiform monophyly is also supported by seven morphological character complexes [1,11,18]. This lineage includes nearly 900 species allocated among six families and is the sister group of the Gobiesocidae (Figure 1), a relationship supported by six morphological characters, one of which is unique to this lineage [19].

Our study supports the monophyly of four of the six blenniiform families as currently construed. The mono-phyly of the Tripterygiidae, Blenniidae, Clinidae and Dactyloscopidae are also well-supported by morphological synapomorphies [1,11,23]. The monophyly of the Chaenopsidae (*sensu* Hastings and Springer, 1994 [37]) and the Labrisomidae are, however, not supported in our analysis (see below).

Within the Blenniiformes, our study indicates that the Tripterygiidae is the sister group to the remaining blennies (Figure 1). This hypothesis was proposed by Springer [11] based on details of the fin rays, scales and cephalic sensory system, and subsequently supported by Springer and Orrell [19] based on details of the branchial musculature. Our analysis also supports the hypothesis of Springer and Orrell [19] based on several features of the dorsal gill-arches and associated muscles that the Blenniidae is the sister group of the remaining four families of blennies (Figure 1). That clade includes the Clinidae, Labrisomidae, Chaenopsidae and Dactylosco-pidae and is referred herein as the “clinioids” (node I; Figures 1 and 4). Relationships within the clinioids are complicated and not entirely consistent with the current nomenclature, as reported by earlier studies based on morphological characters [19], allozyme data [22], and mitochondrial 12S rRNA sequence data [9]. In our analysis, a single species of the labrisomid tribe Cryptotremini (*Calliclinus geniguttatus*) is the sister group of all other clinioids, and the monophyletic family Clinidae is the sister group of the remaining clinioids (Figure 4). Our analysis provided partial resolution of deep relationships within the latter lineage, but with short branch lengths to their most recent common ancestors (Figure 4), implying frequent divergence events (Additional file 2: Figure S1) consistent with a rapid radiation [38]. Bayesian Inference and Maximum Likelihood analyses support another two cryptotremins (*Alloclinus holderi* + *Auchenionchus microcirrhis*) as the sister group of the remaining species, and two “chaenopsids” (*Neoclinus blanchardi* + *Mccoskerichthys sandae*) as the sister group of all others (unresolved in ML) (Figure 4). These low diversity branches are followed by two speciose lineages (node IV), one that includes most labrisomid species (node II; Figure 4), and a second, newly identified clade that includes the Chaenopsidae *sensu stricto* and the Dactyloscopidae (node III; Figure 4).

### *Tripterygiidae*

The Tripterygiidae, comprising 32 genera and 164 species, can be readily distinguished from other blenniiforms by their three-part dorsal fin which lacks the “last” dorsal-fin spine [11,24]. Two subfamilies and eight tribes of triplefins have recently been proposed based on morphological evidence [24,39], but their relationships remain unclear. Recent molecular studies on the phylogenetic relationships of triplefins are restricted to regional studies with limited taxon sampling (e.g. [40-42]) and the same is partially true of our study. We included representatives of four tribes, eight genera and 19 species from the subfamily Tripterygiinae (Table 1 and Additional file 1: Table S1) with an emphasis on eastern Pacific species. Although the monophyly of triplefins is strongly supported by our data, there is a general pattern of low node support values within the family (Figure 2). Our poor resolution of relationships within the Tripterygiidae is likely a consequence of inadequate taxon sampling (19 of 164 species), incomplete data sampling (i.e. missing data for some markers), the presence of numerous insertions and deletions observed in the sequences of TMO-4C4 and RAG1 which complicates phylogenetic analyses [43], and the possible rapid diversification of the group [40]. Clearly much broader taxon sampling and further study will be needed to resolve relationships within the triplefins.

### *Blenniidae*

**Figure 6:**
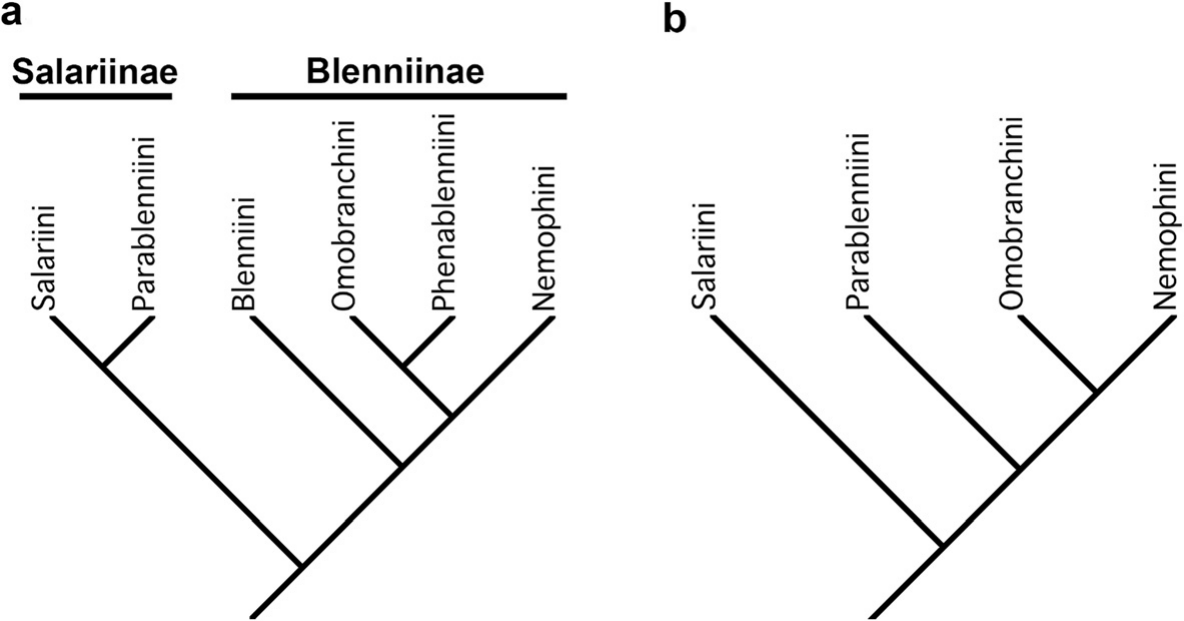
**Phylogenetic relationships of the tribes of the Blenniidae based on (a) morphological evidence**[44,47]**, and (b) molecular data in this study.**

The Blenniidae, comprising 57 genera and 387 species, is the largest and most well-studied lineage of blennies [23]. Combtooth blennies share several unique features including incisiform teeth in a single comblike row on the dentaries and premaxillaries [11,12,44]. While the family is easily distinguished from other blennies, the classification within the Blenniidae has a long and complicated history [23]. The currently recognized tribes Parablenniini and Blenniini were at one time placed within a large, polyphyletic genus *Blennius* by Norman [45] and others. Based on the possession of strongly sutured dentaries, the type of the genus (*Blennius ocellaris* Linnaeus, 1758) was considered distinctive and it, along with a congener and two species of *Spaniblennius,* were designated as the tribe Blenniini [12,46,47]. The remaining “*Blennius*” species were allocated to various other genera and placed in the tribe Parablenniini [48]. The Para-blenniini was hypothesized to be the sister group of the Salariini [44,49], and these together have been recognized as the Salariinae (Figure 6a). The Blenniini was hypothesized to be the sister group of a clade comprising the Omobranchini, Phenablenniini, and Nemophini [47], and these four tribes as a group have been recognized as the subfamily Blenniinae (Figure 6a). The monophyly of each of the six tribes of blenniids, with the notable exception of the Parablenniini, has been confirmed based on morphological characters [23].

We sampled 20 genera and 48 species, representing four of the six tribes and both subfamilies Blenniinae and Salariinae (Table 1 and Additional file 1: Table S1). Two low diversity tribes, the Blenniini with four species and the monotypic Phenablenniini, were not included in our analysis. The monophyly of the four tribes included in our study is supported (Figure 3), as is the monophyly of the Blenniinae. However, the monophyly of the Salariinae (*sensu* Bath, 2001 [49]) is not supported. Instead, we found the Parablenniini to be the sister group of the Blenniinae rather than the Salariini (Figure 6b). However, because our study included few genera of the Parablenniini (2 of 14) and did not include a member of the tribe Blenniini, additional taxon sampling is necessary to more fully resolve the relationships of the major lineages of blenniids.

The Nemophini genera *Meiacanthus, Plagiotremus, Petroscirtus*, and *Xiphasia* form a monophyletic clade that is sister to the Omobranchini genus *Omobranchus* (Figure 3). Within the Nemophini, this analysis provides an alternative phylogeny to the provisional hypothesis proposed by Smith-Vaniz [47] in which *Meicanthus* and *Petroscirtes* together were the sister group of *Plagiotremus*, *Xiphasia,* and *Aspidontis*[47]. In our analysis, *Meiacanthus* and *Plagiotremus* form a strongly supported clade that is the sister group of *Petroscirtus* and *Xiphasia* (Figure 3). Relationships of the three species of *Plagiotremus* included in our study are consistent with those proposed by Smith-Vaniz ([47], Fig 81).

**Figure 7:**
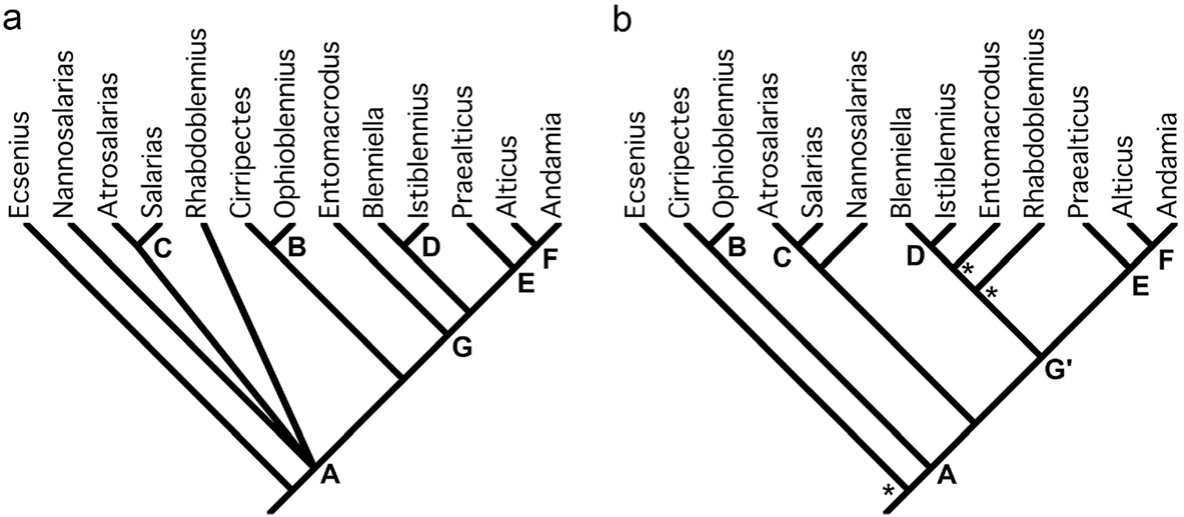
**Generic relationships of the blenniid tribe Salariini. (a)** Hypothesized relationships based on Smith-Vaniz and Springer [50] and updated in Springer and Williams [51]. **(b)** Consensus relationships based on Maximum Likelihood, Maximum Parsimony, and Bayesian Inference in this study. *= node not supported by Maximum Parsimony. Nodes labeled with the same letters **(A**-**G)** in both **(a)** and **(b)** are identical. Node G’ in **(b)** is different from node G in **(a)** by including *Rhabdoblennius*.

The Salariini is the largest blenniid tribe with well over 200 species and is characterized by unique features of the premaxilla, pharyngeal arches and pectoral girdle [23]. Within the Salariini, Williams [44] recognized two lineages, the *Salarias* group and the *Rhabdoblennius* group, the former with a highly modified dentary and numerous premaxillary teeth [49], and the latter lacking known synapomorphies. In the present study, we sampled thirteen of the 28 Salariini genera. All genera with more than one included species were found to be monophyletic except *Andamia* and *Salarias* (Figure 3). However, the lineages hypothesized by Williams [44] were not supported as the only genus of the *Rhabdoblennius* group available for our study (*Rhabdoblennius*) was nested within the *Salarias* group (Figure 3). Otherwise, the generic relationships recovered (Figure 7b) are similar to, and better resolved, than morphologically-based hypotheses [50,51] (Figure 7a). Analyses of both character types (Figure 7a and 7b) support the genus *Ecsenius* as the sister group of the remaining salariins (node A), *Cirripectes* and *Ophioblennius* as sister groups (node B), *Atrosalarias* and *Salarias* as sister groups (node C), *Blenniella* and *Istiblennius* as sister groups (node D), and *Praealticus* as the sister group (node E) of a clade including *Andamia* and *Alticus* (node F). In the morphology-based tree (Figure 7a), the *Praealticus* clade shares a most recent common ancestor with *Blenniella* and *Istiblennius*, that is the sister group of the genus *Entomacrodus* (node G). However, the molecular analysis includes *Entomacrodus*, as well as *Rhabdoblennius,* in this clade (node G'). Finally in the morphological hypothesis, *Cirripectes* and *Ophioblennius* (node B) are the sister group of node G, while in the molecular hypothesis, the *Salarias* clade (node C + *Nannosalarias*) is the sister group of node G’.

Only two out of the fourteen Parablenniini genera (*Hypsoblennius* and *Parablennius)* are included in our study and these form a monophyletic group (Figure 3). The six *Hypsoblennius* species show incongruent relationships from those suggested by Bath [52] in several respects.

### *Clinidae*

The monophyly of the Clinidae, comprising 26 genera and 85 species, is supported by the presence of cycloid scales with radii in all fields and a cordlike ligament extending from the ceratohyal to the dentary [11,14,53]. Three tribes are recognized within this family [14,53] and a hypothesized relationship of the Myxodini as the sister group of the Clinini + Ophiclinini is based on the latter two sharing the reproductive pattern of internal fertilization with males possessing an intromittent organ [53]. Our study includes representatives of the Clinini and Myxodini and the monophyly of each is well supported (Figure 4). Species relationships within the Myxodini genus *Gibbonsia* (Figure 4) are congruent with the previously hypothesized relationships based on 40 allozyme loci [54], but conflict with a more recent study with expanded taxon sampling based on the same allozyme loci [22] and 12S rRNA data [9]. Within the Clinini, the Australian genera *Heteroclinus* and *Cristiceps* form a well-supported sister group to the South African genera *Clinus*, *Muraenoclinus*, *Blennophis*, and *Pavoclinus* (Figure 4)*.*

### *Labrisomidae*

The monophyly of the Labrisomidae has long been questioned because of the lack of any supporting morphological characters [11,19] or molecular evidence [9,22]. Also, relationships among the five included tribes remain unclear [1]. Our study includes representatives of all five hypothesized tribes and 10 of the 14 genera (Table 1 and Additional file 1: Table S1). The only genera not included are the Eastern Pacific deepwater (> 20 m depth) genus *Cryptotrema* with two species (Cryptotremini; [14,55]), the recently described monotypic *Cottoclinus* (Mnierpini; [56]), and two rare and poorly known genera of uncertain relationships, *Nemaclinus* with one species [57] and *Haptoclinus* with two species [21,58]. The present study thus provides the most thorough investigation to date of the phylogenetic relationships of members of this family.

In our analysis, a monophyletic Labrisomidae (node II; Figure 4) is recovered with the exclusion of the Crypto-tremini and the inclusion of *Stathmonotus*. In our BI analysis, *Stathmonotus* is the sister group of the Para-clinini, and that clade is the sister group of a clade including the Starksiini and Labrisomini (Figure 4). However, relationships among the major lineages of labrisomids have strong nodal support only from the BI analysis.

The tribe Cryptotremini is the only exclusively temperate group historically allocated to the Labrisomidae while all others are primarily tropical. This tribe was first described by Hubbs [14] who included the northeastern Pacific genera *Alloclinus* and *Cryptotrema.* The Cryptotremini was later expanded with the addition of two southeastern Pacific genera (*Auchenionchus* and *Calliclinus*), although their inclusion was based on the plesiomorphic condition of branched caudal-fin rays [59]. The caudal-fin rays are unbranched in all other labrisomids but branched in the Tripterygiidae, most Blenniidae and some Dactyloscopi-dae. None of the analytical methods used in our study support the monophyly of the Cryptotremini (Figure 4). Instead the genus *Calliclinus* is the sister group of all other clinioids, the northern genus *Alloclinus* is grouped with the southern genus *Auchenionchus,* and that clade is the sister group of the clinioids exclusive of the Clinidae and *Calliclinus*. While support values for many of these relationships are not strong, the evolutionary position of cryptotremins as early branching lineages of a diverse clade including the labrisomids was also suggested by morphological [59] and allozyme data [22]. Additional study of cryptotremin blennies should provide a clearer picture of clinioid relationships.

The tribe Labrisomini, including *Labrisomus* and *Malacoctenus,* is not defined by morphological synapomorphies [60]. However, the monophyly of this tribe was supported by mitochondrial 12S rRNA data from two *Labrisomus* and two *Malacoctenus* species [9]. With a much broader taxon sampling and multiple genetic markers in our study, the Labrisomini is recovered as monophyletic (Figure 4), but the monophyly of the genus *Labrisomus* as currently construed is not supported. Based on our concatenated molecular data, the seven *Labrisomus* species are divided into two lineages (Figure 4). The first lineage, represented in our study by *L. nuchipinnis* and *L. xanti* (both previously allocated to the subgenus *Labrisomus*; Table 3; [14,60,61]) is the sister group of the genus *Malacoctenus.* The second *Labrisomus* lineage includes two sub-lineages for which generic names are available. One includes *L. nigricinctus* and *L. striatus*, species assigned to the subgenus *Brockius* (Table 3; [60,61]), while the other includes *L. haitiensis*, *L. bucciferus* and *L. guppyi*, species previously assigned to the subgenus *Gobioclinus* (Table 3; [60]). Members of the subgenus *Gobioclinus* can be distinguished from other *Labrisomus* species by the presence of palatine teeth, several of which are considerably larger than those on vomer. Members of the subgenus *Brockius* have fewer scales in the lateral line compared to other species currently allocated to *Labrisomus,* and all posterior lateral-line scales have the anterior pore of the canal exposed [60]. Although the present study includes only seven of the twenty-one *Labrisomus* species, these three *Labrisomus* clades based on molecular data are congruent with Hubbs’ [14] and Springer’s [60] three subgenera. As Springer ([60]; p. 422) suspected, “I feel certain that some systematists would relegate each of the above subgenera (*Labrisomus*, *Brockius*, and *Gobioclinus*) to the rank of genus, as the differences separating them are trenchant.” Our study includes the type species of all three subgenera and confirms Springer’s assessment. Thus we recognize these three as full genera with the genus *Brockius* Hubbs, 1953 (type species = *Brockius striatus* Hubbs, 1953; [61]) the sister group of the genus *Gobioclinus* Gill, 1860 (type species = *Clinus gobio* Valenciennes, 1836; [62]) and the restricted genus *Labrisomus* (type species = *Clinus pectinifer* Valenciennes, 1836, a synonym of *Clinus nuchipinnis* Quoy and Gaimard, 1824) the sister group of the genus *Malacoctenus*. Species placed in each of these genera based on morphological criteria are listed in Table 3.

**Table 3:** **Species allocated to three genera previously placed in the genus *Labrisomus***

	
*Labrisomus* Swainson, 1837	*Labrisomus conditus* Sazima, Carvalho-Filho, Gasparini and Sazima, 2009 ^a^
	*Labrisomus cricota* Sazima, Gasparini and Moura, 2002 ^a^
	*Labrisomus fernandezianus* (Guichenot, 1848)^#^
	*Labrisomus jenkinsi* (Heller and Snodgrass, 1903)
	*Labrisomus multiporosus* Hubbs, 1953
	*Labrisomus nuchipinnis* (Quoy and Gaimard 1824)*^+^
	*Labrisomus philippii* (Steindachner, 1866)
	*Labrisomus pomaspilus* Springer and Rosenblatt, 1965 ^a^
	*Labrisomus socorroensis* Hubbs, 1953
	*Labrisomus wigginsi* Hubbs, 1953
	*Labrisomus xanti* Gill, 1860 ^+^
	
*Brockius* Hubbs, 1953	*Brockius albigenys* (Beebe and Tee-Van, 1928)
	*Brockius nigricinctus* (Howell Rivero, 1936) ^+^
	*Brockius striatus* (Hubbs, 1953)* ^+^
	
*Gobioclinus* Gill, 1860	*Gobioclinus bucciferus* (Poey, 1868) ^+^
	*Gobioclinus dendriticus* (Reid, 1935)
	*Gobioclinus filamentosus* (Springer, 1960)
	*Gobioclinus gobio* (Valenciennes 1836)*
	*Gobioclinus guppyi* (Norman, 1922) ^+^
	*Gobioclinus haitiensis* (Beebe and Tee-Van, 1928) ^+^
	*Gobioclinus kalisherae* (Jordan, 1904)

^a^ = placement based on details in the original species description; * = type species; ^#^ = placement tentative; ^+^ = included in this study.

Ten of the twenty-one *Malacoctenus* species are included in our study and these form a well-supported clade that is the sister group of *Labrisomus sensu stricto* (Figure 4). Our study recovered one well-supported lineage within *Malacoctenus* comprising five Eastern Pacific species. However, greater taxon sampling, especially of Caribbean species, is needed to resolve the relationships within this speciose genus.

The monophyly of the tribe Starksiini, including *Starksia* and *Xenomedea*, is supported in our analysis (Figure 4). Members of this lineage reportedly share internal fertilization and a modification of the first anal-fin spine that functions as an intromittent organ in males [14,63]. However, a recent study indicates that reproductive modes vary within this lineage and not all *Starksia* species exhibit internal fertilization [64].

The two genera currently included in the tribe Paraclinini, *Paraclinus* and *Exerpes*, share the unique characters of a spine on the posterior margin of the opercle and 0–2 segmented dorsal-fin rays [14,65,66]. Results from our study support the monophyly of the tribe (Figure 4), although the monotypic genus *Exerpes* is nested within *Paraclinus*, rendering the genus *Paraclinus* paraphyletic. *Exerpes asper* can be distinguished from members of the genus *Paraclinus* in having greatly prolonged snout, no cirri on the nape or eye, scales in the anterior segment of the lateral line consisting of a pore at each end of a tube, and by the absence of the suborbital lateral-line canal [14]. However, this species shares several features such as an elongate snout with selected *Paraclinus* species, especially *Paraclinus infrons*[66]. Additional study of relationships within this lineage are needed, but based on our findings and those of Brooks [66], we synonomize the genus *Exerpes* Jordan and Evermann, 1896 with *Paraclinus* Mocquard, 1888.

The phylogenetic relationships of the seven species of small (< 55 mm SL), cryptic, eel-like blennioids of the genus *Stathmonotus* have been controversial for many years [37]. Jordan [15] considered *Stathmonotus* to be closely related to chaenopsids based on both groups lacking scales. However, Springer [67] included the scaled species *Auchenistius stahli* in *Stathmonotus*, and suggested that their affinities were with *Paraclinus*, not chaenopsids. Consequently, *Stathmonotus* was included in the generalized blenniiform family Labrisomidae [68,69]. Based on six morphological synapomorphies, Hastings and Springer [37] placed *Stathmonotus* back in the Chaenopsidae as the sister group of the Chaenopsinae (= Chaenopsidae of Stephens, 1963; [70]). The present study based on molecular data confirms the distinctiveness of *Stathmonotus*, but the BI analysis supports its relationship as the sister group of the Paraclinini (Figure 4). *Stathmonotus* is similar to *Paraclinus* in post-cranial morphology, especially in having the dorsal fin comprised entirely (or mostly in the case of some *Paraclinus* species) of robust spines. However, it is similar to chaenopsids in cranial morphology and parsimony analysis of morphological characters places *Stathmonotus* with the chaenopsids because more characters are evident within the cranial region [37]. These observations, with different suites of characters shared with disparate lineages, are consistent with a hybrid origin for this enigmatic taxon [71], but this hypothesis remains speculative, requiring further study. Given its morphological distinctiveness [37], we recommend recognizing the tribe Stathmonotini within the Labrisomidae.

### *Neocliniini*

Stephens [70] hypothesized the close relationship of the temperate hole-dwelling genus *Neoclinus* with the tropical chaenopsid blennies. This relationship was given further credence by the discovery of the tropical species *Mccoskerichthys sandae* that resembles *Neoclinus*, but also shares several morphological features (e.g., medially fused nasal bones) with chaenopsids [72]. The recent placement of *Neoclinus* and *Mccoskerichthys* in the lineage with the chaenopsids based on a parsimony analysis of morphological features [37] is not supported in our study and implies significant morphological convergence associated with the hole-dwelling lifestyles of these fishes. The sister-group relationship between *Neoclinus* and *Mccoskerichthys* is well supported in our study, thus we recommend their designation as the Neocliniini. However, their placement as the sister group of the clinioids exclusive of the Cryptotremini and Clinidae (Figure 4) is only weakly supported by Bayesian Inference. Further study is required to determine their position within the Blenniiformes.

### *Chaenopsidae*

In our analysis, the Chaenopsidae is monophyletic with the exclusion of *Stathmonotus*, *Neoclinus* and *Mccoskerichthys*, three genera allocated to this group by Hastings and Springer [37]. This result resurrects the definition of the Chaenopsidae *sensu* Stephens [70,73]. This clade is well-supported by morphological features and relationships within it were recently analyzed by Lin and Hastings [74] using a combination of molecular and morphological characters. Results from the present analysis that included a much broader selection of outgroup taxa but fewer ingroup species, are largely congruent with those of Lin and Hastings [74] except for species relationships within *Acanthemblemaria*, a group recently studied in greater detail [75].

### *Dactyloscopidae*

The dactyloscopids, also known as sand stargazers, are a distinctive group with several known morphological syanpomorphies, many of which are associated with their sand or gravel dwelling behaviors [1,11,36]. In our study, only four out of the 48 dactyloscopid species were included and they formed a well-supported monophyletic group based on our molecular data (Figure 4).

### Unresolved issues in blenniiform relationships

While our analysis clarifies several aspects of the relationships of the Blenniiformes, several significant questions remain. Relationships within the Tripterygiidae are not well understood and our study, with limited taxon sampling, contributes little to this issue. Inclusion of taxa from the southern Pacific Ocean, especially from New Zealand and southern Australia where this group is especially diverse [24,39-42], is needed to resolve relationships within the triplefins. Within the Blenniidae, the relationships of the low diversity, but morphologically distinctive, tribes Blenniini and Phenablenniini need further study. Also, the monophyly of the Parablenniini has not been confirmed although its reality and its relationships to other blenniids may have significant bearing on relationships within the combtooth blennies [23]. Several significant questions remain regarding relationships among the lineages here termed the clinioids (node I; Figures 1 and 4). Chief among these is the relationships of the species currently allocated to the Cryptotremini. This low diversity group of relatively generalized blennies is apparently not monophyletic and in our analysis, its members are variously placed as the sister group of speciose clades of blennies. Morphological convergence in lifestyle and associated morphological features appear to have confounded past morphologically-based analyses of blenny relationships, especially among the tube-dwelling lineages. Also, relationships of the enigmatic worm blennies, genus *Stathmonotus,* remain unclear as they have for decades. This study supports other recent studies [32,37] in hypothesizing a sister-group relationship of the Dactyloscopidae and Chaenopsidae (node III; Figure 4). Preliminary study indicates possible morphological features uniting these two (PAH, personal observations), but characterization of this clade may have been confounded by the inclusion of the apparently unrelated fishes of the genera *Neoclinus, Mccoskerichthys* and *Stathmonotus* within the Chaenopsidae. Finally, our understanding of species level-relationships within most lineages included herein suffers from poor taxon sampling. Thus relationships recovered in our analyses within speciose genera for which we had few representative species should be considered tentative.

Because of these and other significant issues, we are reluctant to recommend major changes to the higher-level nomenclature of the Blenniiformes at this time. While progress has been made, these and other remaining challenges in resolving blenniiform relationships will require incorporation of additional molecular markers for these and additional taxa, and importantly inclusion of morphological features in a total evidence analysis.

### Biogeography of the Blenniiformes

Our mean estimated divergence time for the Blenniiformes, 60.3 mya (Additional file 2: Figure S1), is similar to recent estimates from other studies of ray-finned fishes that place the origin of the Blenniiformes at around 60 [76] to 68 mya [35]. Our study also implies the origin of a largely Neotropical clade of over 240 species (node IV) of the Labrisomidae, Chaenopsidae and Dactyloscopidae at approximately 37.6 mya, somewhat more recent than other estimates of 40 mya [35] and 48 mya [34] for the same clade.

The majority of species in the families Blenniidae and Tripterygiidae occur in the tropical IndoPacific with various other lineages of these families found in the Neotropics and temperate waters of the world [6,77,78]. Our analysis implies an origin of the Blenniiformes in the present-day IndoPacific region (Figure 5, Additional file 3: Figure S2), consequently in the Tethys Sea at approximately 60 mya. The origin of the largely Neotropical clade including the Labrisomidae, Chaenopsidae and Dactyloscopidae (node IV) at approximately 37.6 mya is coincident with the increasing opening of the Atlantic Ocean and separation of the New World and Old World land masses, but well before the closing of the Tethys Sea corridor at 12–18 mya [79,80]. This result supports the findings of Bellwood and Wainwright [81] that the east and west Tethyan reef-fish faunas had diverged well before the terminal Tethyan event.

Interestingly, the intervening blenniiform clades between the primarily east and west Tethyan groups are almost exclusively temperate in distribution (Figure 5, Additional file 3: Figure S2). This finding implies either an evolutionary pathway across temperate coastal areas between increasingly separate east and west tropical regions of the Tethys Sea, or more likely, the subsequent restriction of the intervening clades to temperate refugia [82]. These groups include low-diversity lineages currently included in the paraphyletic Cryptotremini (e.g., *Calliclinus*, *Auchenionchus*, *Alloclinus* and *Cryptotrema*) [59] and the genus *Neoclinus*[6,61], as well as the relatively speciose Clinidae, with 85 species, that has undergone significant diversification within distant temperate regions of the world [78]. The occurrence of these related clades in both northern and southern temperate regions indicates that they have successfully crossed intervening tropical regions during their evolutionary history [82,83]. Better resolution of relationships and timing of divergence of these largely temperate lineages [6,77,78] is an important key to reconstructing the biogeographic history of the Blenniiformes in greater detail.

Other Neotropical lineages of blenniiforms include the salariin blenniid genera *Ophioblennius* and *Scartichthys* (the latter not included in our study), part or parts of the Parablenniini [48,84], and one or more lineages within the Tripterygiidae [6,24], as well as the Gobiesocini within the sistergroup of blenniiforms, the Gobiesocidae [85]. A more fully resolved phylogeny of these groups with increased taxon sampling is needed to determine which, if any, of these lineages may have diverged coincident with the Neotropical blenniiforms (node IV). A few species of blennies from other primarily IndoPacific clades have invaded the Neotropics including species of the salariin genus *Entomacrodus*[86] and a single species of the nemophin blenniid genus *Plagiotremus*[47]. These species are well-nested within IndoPacific clades (Figure 5), supporting the hypotheses that they dispersed to the Neotropics after the origin of their respective genera in the IndoPacific region [6]. Similarly, the few species of primarily Neotropical clades occuring in the eastern Atlantic (e.g., species of the labrisomid genera *Labrisomus* and *Malacoctenus*; [6,87]), and members of the temperate genus *Neoclinus* in the northwestern Pacific [88], likely represent relatively recent dispersal events from their regions of origin.

## Conclusions

In this study we reconstruct the phylogeny of the Blenniiformes with significantly broader taxon sampling (150 blenniiform species), substantially more genetic information (one mitochondrial and four nuclear markers), and more strategic outgroup representation (Gobiesocidae, Opistognathidae, and Grammatidae) than in previous studies. Progress has been made in resolving the blenniiform evolutionary relationships especially at the inter-familial level and within the Labrisomidae. Several nomenclatural changes are proposed especially in the “clinioid” clade. Examination of global distributions of blenny genera included in our analysis and estimation of divergence times imply an origin of the Blenniiformes in the present-day IndoPacific region, consequently in the Tethys Sea around 60 million years ago. A large and diverse Neotropical clade (node IV, Figures 1, 4, and 5) arose around 37.6 million years ago with intervening lineages largely restricted to temperate regions.

## Methods

### Taxon sampling

Molecular data for 158 terminal taxa were collected. Additional file 1: Table S1 details the included species, collection localities and deposition of voucher specimens. Our taxon sampling included representatives of all six blenniiform families (Table 1): Tripterygiidae (18 species), Blenniidae (48), Labrisomidae (36), Chaenopsidae (30), Clinidae (14), and Dactyloscopidae (4), as well as the outgroups Gobiesocidae (6), Opistognathidae (1), and Grammatidae (1). Currently recognized lineages within the blenniiform families were sampled with representative species where available (Table 1). Tissue samples were from the Marine Vertebrate Collection at Scripps Institution of Oceanography (SIO), the University of Kansas Natural History Museum (KU), the Biodiversity Research Museum at Academia Sinica (ASIZP), Taiwan and the Australian Museum (AM).

### DNA extraction, amplification and sequencing

Total genomic DNA was extracted from muscle tissue with a Qiagen (Chatsworth, CA) QIAquick Tissue Kit following the manufacturer’s instructions. DNA sequences of one mitochondrial DNA marker, Cytochrome C Oxidase I (COI), and four nuclear markers, TMO-4C4, RAG1, Rhodopsin and Histone H3, were obtained. In addition to the primers used in a recent publication on chaenopsid phylogeny [74], six new primers were designed for amplifying PCR products across this broad sample of taxa: two extended inside primers from TMO-F3 and TMO-R3 for TMO-4C4, TMO-F4 5′-GGTGAAGTGGTTCTGCAACA-3′ and TMO-R4 5′-GCYGTGTACTCNGGRATRGT-3′; two gobiesocid-specific inside primers for RAG1, Rag-GoF 5′-TTCCTCGATCATTTAGTTTCCA-3′ and Rag-GoR 5′-GAAGGGCTTGGAGGAAACTC-3′; two blenniiform-specific inside primers for Rhodopsin, Rhod-BleF 5′-CGTCACCCTCGAACACAAGAA-3′ and Rhod-BleR 5′-GTTGTAGATGGAGGAACTCTT-3′. The PCR was performed on a Mastercycler EP Gradient S (Eppendorf, Hamburg, Germany) with the following conditions: 94°C for one minute for initial denaturing, 35 cycles of 94°C for 30 sec, 52-56°C for 45 seconds, and 72°C for 45 sec, followed by 72°C for 5 minutes as the final extension. Resulting amplicons were purified with Exonuclease I (20 U/μl, New England Biolabs) and Shrimp Alkaline Phosphatase (1 U/μl, Roche) in order to remove single-stranded DNA and unincorporated dNTPs. Sequencing was done in both directions with the amplification primers and DYEnamicTM ET dye terminator sequencing kit on an automated MegaBACE™ 500 DNA sequencer (Amersham Biosciences Corp., Piscataway, NJ).

### DNA sequence alignment, partition, and analysis

Sequences were assembled, edited, and aligned with no gaps interrupting reading frames based on translated protein sequences in Geneious [89]. Nucleotide sequences were checked on the NCBI database (http://www.ncbi.nlm.nih.gov/) for possible stop codons as an indication of pseudogenes. Because all five genetic markers are protein-coding genes, the rapidly evolving third codon positions were partitioned from the slower evolving first and second positions in Bayesian and likelihood analyses, resulting in ten total partitions [90]. To avoid base frequency deviations across taxa which can potentially mislead phylogenetic reconstruction [91], the chi-square test of base frequency homogeneity across taxa was executed for each partition using PAUP 4.0b10 [92]. The Akaike Information Criterion (AIC) [93] implemented in jModelTest v0.1.1 [94,95] was used to select the best-fit evolutionary model for each partition (Table 2). Likelihood calculations were carried out for 88 models, including 11 substitution schemes, equal or unequal base frequencies, a proportion of invariant sites (I), and rate variation among sites with four rate categories (G) on a BIONJ-JC fixed tree.

### Phylogenetic analysis

Maximum likelihood (ML), Bayesian inference (BI), and maximum parsimony (MP) analyses were conducted to reconstruct the phylogenetic relationships. Maximum likelihood tree searching was conducted in Garli 2.0 [96] under the CIPRES Science Portal v2.2 [97]. Best-fit evolutionary models selected by jModeltest were applied to the ten genetic partitions (five genes for first + second and third codon positions) (Table 2). If a selected model could not be implemented in Garli, the least complex model that included all of the parameters of the selected model was used instead. Therefore, the General Time Reversible model with gamma rate heterogeneity model (GTR+G) was selected for the third codon position of COI, and the GTR+I+G model [98] was selected for all other partitions. Twelve replicates were run to find the tree topology with the best likelihood. The setting of 1,000 replicate bootstrap analysis was identical to the above, except the number of generations without topology improvement required for termination (genthreshfortopoterm) was reduced from 20,000 (default) to 10,000 to reduce the running time as suggested in the manual [96].

Bayesian Metropolis coupled Markov chain Monte Carlo (MCMC) estimation of the phylogeny was carried out using MrBayes v3.1.2 [99] under the CIPRES Science Portal. The dataset was partitioned and assigned evolutionary models as in the ML analyses. A partitioned mixed-model analysis was applied and all model parameter values were “unlinked” among partitions [100]. In all analyses, the average substitution rates (prset ratepr = variable) and model parameters including the branch lengths within the tree (unlink brlens) were allowed to vary among partitions. Two simulated independent runs were performed for 10 million generations each and starting from different random trees. Each run comprised four chains (one cold and three heated) and was sampled every 1,000 generations. The sampled parameter values from Bayesian MCMC were evaluated in Tracer v1.4 (http://beast.bio.ed.ac.uk/Tracer) and the generations before reaching a plateau were discarded as burnin. Trees from the stationary phase of the two runs were then pooled by LogCombiner v1.5.4 [101] and the 50% majority tree was exported by Mesquite v2.73 [102]. Assigning this tree as the target tree, the posterior probability of each node and the mean branch lengths were calculated with TreeAnnotator v1.5.4 [101].

Maximum parsimony analyses were conducted using PAUP 4.0b10. Heuristic searches were performed using tree bisection reconnection (TBR) with branch-swapping from 1,000 random-addition-sequence replicates to avoid entrapment in local optima. All nucleotide sites were equally weighted and gaps were treated as missing characters. Nonparametric node supports for trees were estimated with 1,000 heuristic searches (maxTree = 500) starting with 10 random addition sequence replicates.

### Molecular dating

Divergence times of the blenniiform lineages sampled in our study were estimated with a relaxed molecular clock analysis [103]. Relative divergence times of nodes were estimated in BEAST v1.7.2 assuming an uncorrelated lognormal model (UCLN) of rate variation among branches in the tree and a Yule-process-speciation prior of the branching rate [101,103]. As in the maximum likelihood and Bayesian analysis described before, the same ten data partitions and molecular evolutionary models were applied to estimate the posterior density of relative divergence times. With TreeEdit v1.0a10 (http://tree.bio.ed.ac.uk/software/treeedit/), the best ML tree from Garli was converted to an ultrametric tree assuming the origin of the Blenniidae as 41 million years old as estimated from a bony fish phylogeny including 21 molecular markers and 1410 fish taxa [34] and assigned as the starting tree. We did not incorporate the putative blenniid fossil from the Monte Bolca formation (50 mya; [104]), because its identity as a blenniid is in doubt ([11,105], Springer, pers. comm). Three independent MCMC analyses were each run for 40 million generations, sampled every 1000 generations, and discarding the first 10% of samples, resulting in acceptable mixing as determined by Tracer. These three runs were combined to obtain an estimate of the posterior distribution. The posterior probability density of the combined tree and log files was summarized on a maximum clade credibility tree with TreeAnnotator.

### Biogeographic analysis

The general distributions of blenniiform genera with representatives included in our analysis were scored as either tropical IndoPacific (I), Neotropical (N), or temperate (T; Additional file 4: Table S2). Genera with species present in more than one of these were scored as polymorphic except where phylogenetic evidence implies recent dispersal into another region after the origin of the genus. For example, *Plagiotremus* (Blenniidae) was scored as IndoPacific because the single Neotropical species (*P. azaleus*) is nested within an otherwise entirely IndoPacific clade ([6,47]; Figure 3).

We used both parsimony implemented in Mesquite and Bayesian Binary MCMC (BBM) methods implemented in Reconstruct Ancestral States in Phylogenetics v2.1 beta (RASP; [106]) to reconstruct geographical areas at nodes in the phylogeny. The genus–level topology of the best ML tree was used for the reconstruction. The BBM analysis was conducted with estimated character state frequencies (F81) and gamma-distributed rate variation between sites. The root distribution was set to null (i.e. the outgroup is assigned to a new area where none of the ingroup taxa occur). Two independent runs of 10 chains with a temperature of 0.1 were run for one million generations, sampled every 100 generations, and the first 2,500 samples were discarded. A distance between runs of less than 0.01 was used as an indication of convergence.

## Competing interests

The authors declare that they have no competing interests.

## Authors’ contributions

HCL and PAH conceived the study, conducted analyses and wrote the manuscript. Both authors read and approved the final manuscript.

## Supplementary Material

Additional file 1: Table S1 Abbreviations, voucher numbers, localities, sample IDs, and Genbank accession numbers for 158 terminal taxa used in this phylogenetic analysis. Click here for file

Additional file 2: Figure S1 Posterior maximum clade credibility relative time tree of blenniiform species inferred from a relaxed molecular clock analysis using BEAST. Branches are scaled to age estimates. Bars at nodes reflect the 95% highest posterior density of the age estimates. Click here for file

Additional file 3: Figure S2 Ancestral distribution at each node of the blenniiform phylogeny estimated by MP analysis implemented in Mesquite. Click here for file

Additional file 4: Table S2 Biogeographical distribution of blenniiform genera. Click here for file
