# Supplementary material for: Phylogeny and biogeography of a shallow water fish clade (Teleostei: Blenniiformes)
Source: BMC Evol Biol. 2013 Sep 25;13:210. doi: 10.1186/1471-2148-13-210 (PMC3849733; doi:10.1186/1471-2148-13-210)
Supplement: Additional file 1: Table S1 — Abbreviations, voucher numbers, localities, sample IDs, and Genbank accession numbers for 158 terminal taxa used in this phylogenetic analysis. [file 1471-2148-13-210-S1.doc]

­Table S1

Abbreviations (Abb.), voucher numbers, localities, sample IDs, and Genbank accession numbers for 156 terminal taxa used in this phylogenetic analysis. SIO: Marine Vertebrate Collection of Scripps Institution of Oceanography, KU: Natural History Museum, University of Kansas, ASIZP: Biodiversity Research Museum at Academia Sinica, Taiwan, AM: Australian Museum, C: Gift from S. von der Heyden, University of Stellenbosch, South Africa. Numbers in parentheses are the sample size for each family.

| Taxon | Voucher ID | Abb. | Locality | COI | TMO-4C4 | Rag-1 | Rhodopsin | Histone H3 |
| --- | --- | --- | --- | --- | --- | --- | --- | --- |
| **Family Tripterygiidae (18)** |  |  |  |  |  |  |  |  |
| *Axoclinus storeye* | SIO 01-49-1 | Axca | Bahia Conejas, Mexico | HQ168528 | HQ169003 | - | HQ168879 | HQ168640 |
| *Axoclinus lucillae* | SIO 01-164-1 | Axlu | Taboguilla, Panama | HQ168529 | HQ169004 | HQ168761 | HQ168880 | HQ168641 |
| *Axoclinus nigricaudus* | SIO 02-16-1 | Axni | La Paz, Mexico | HQ168530 | HQ169005 | HQ168762 | HQ168881 | HQ168642 |
| *Cremnochorites capensis* | KU 6473 | Crca | Port Alfred, South Africa | HQ168531 | HQ169006 | HQ168763 | HQ168882 | HQ168643 |
| *Crocodilichthys gracilis* | SIO 06-54-1 | Crgr | Bahia de los Angeles, Mexico | HQ168532 | HQ169007 | - | HQ168883 | HQ168644 |
| *Enneanectes altivelis* | KU 225 | Enal | Carrie Bow Cay, Belize | HQ168533 | HQ169008 | HQ168764 | HQ168884 | HQ168645 |
| *Enneanectes boehlkei* | KU 166 | Enbo | Carrie Bow Cay, Belize | HQ168534 | HQ169009 | - | HQ168885 | HQ168646 |
| *Enneanectes glendae* | SIO 01-182-1 | Ensp | Puerto Vallarta, Mexico | HQ168535 | HQ169010 | HQ168765 | HQ168886 | HQ168647 |
| *Enneanectes macrops* | SIO 01-182-1 | Enma | Puerto Vallarta, Mexico | HQ168536 | HQ169011 | HQ168766 | HQ168887 | HQ168648 |
| *Enneanectes pectoralis* | KU 167 | Enpe | Carrie Bow Cay, Belize | HQ168537 | HQ169012 | HQ168767 | HQ168888 | HQ168649 |
| *Enneanectes reticulatus* | SIO 02-16-1 | Enre | La Paz, Mexico | HQ168538 | HQ169013 | - | HQ168889 | HQ168650 |
| *Enneanectes carminalis* | SIO 01-164-1 | Ense | Taboguilla, Panama | HQ168539 | HQ169014 | HQ168768 | HQ168890 | HQ168651 |
| *Enneapterygius abeli* | KU 7026 | Enab | Mahe, Seychelles | - | HQ169015 | HQ168769 | HQ168891 | HQ168652 |
| *Enneapterygius gruschkai* | KU 7150 | Engr | Mahe, Seychelles | HQ168540 | HQ169016 | HQ168770 | HQ168892 | HQ168653 |
| *Enneapterygius tutuilae* | KU 5483 | Entu | Saipan, CNMI | HQ168541 | HQ169017 | - | HQ168893 | HQ168654 |
| *Helcogramma ellioti* | KU 801 | Heel | Tongatapu, Tonga | HQ168542 | HQ169018 | HQ168771 | HQ168894 | HQ168655 |
| *Helcogramma fuscopinna* | KU 7025 | Hefu | Mahe, Seychelles | HQ168543 | HQ169019 | - | HQ168895 | HQ168656 |
| *Lepidonectes corallicola* | SIO 00-154-1 | Leco | Islas Galapagos | HQ168544 | HQ169020 | HQ168772 | HQ168896 | - |
|  |  |  |  |  |  |  |  |  |
| **Family Blenniidae (48)** |  |  |  |  |  |  |  |  |
| *Alticus saliens* | KU 7531 | Alsa | Pingtung, Taiwan | HQ168546 | HQ169022 | HQ168774 | HQ168898 | HQ168657 |
| *Andamia reyi* | KU 7517 | Anre | Pingtung, Taiwan | HQ168547 | HQ169023 | HQ168775 | HQ168899 | HQ168658 |
| *Andamia tetradactylus* | KU 7519 | Ante | Pingtung, Taiwan | HQ168548 | - | HQ168776 | HQ168900 | HQ168659 |
| *Atrosalarias fuscus* | KU 4125 | Atfu | Viti Levu, Fiji | HQ168549 | - | HQ168777 | - | HQ168660 |
| *Blenniella chrysospilos* | KU 4180 | Blch | Viti Levu, Fiji | - | HQ169024 | HQ168778 | HQ168901 | HQ168661 |
| *Blenniella cyanostigma* | KU 5539 | Blcy | Saipan, CNMI | HQ168551 | HQ169025 | HQ168779 | HQ168902 | HQ168662 |
| *Cirripectes castaneus* | KU 7056 | Cica | Cap Ternay, Seychelles | HQ168552 | HQ169026 | HQ168780 | HQ168903 | HQ168663 |
| *Cirripectes filamentosus* | KU 6917 | Cifi | Mahe, Seychelles | HQ168553 | - | HQ168781 | HQ168904 | HQ168664 |
| *Cirripectes polyzona* | KU 4315 | Cipo | Viti Levu, Fiji | HQ168554 | HQ169027 | HQ168782 | - | HQ168665 |
| *Cirripectes quagga* | KU 800 | Ciqu | Tongatapu, Tonga | HQ168555 | HQ169028 | HQ168783 | HQ168905 | HQ168666 |
| *Cirripectes stigmaticus* | KU 686 | Cist | Tongatapu, Tonga | - | - | HQ168784 | HQ168906 | HQ168667 |
| *Ecsenius midas* | KU 7206 | Ecmi | Ils du Nord, Seychelles | HQ168557 | HQ169030 | HQ168785 | HQ168907 | - |
| *Ecsenius nalolo* | KU 7095 | Ecya | Mahe, Seychelles | HQ168558 | HQ169031 | HQ168786 | HQ168908 | HQ168668 |
| *Ecsenius opsifrontalis* | KU 5576 | Ecop | Saipan, CNMI | HQ168559 | HQ169032 | HQ168787 | HQ168909 | HQ168669 |
| *Ecsenius pardus* | KU 4090 | Ecpa | Viti Levu, Fiji | - | HQ169033 | HQ168788 | HQ168910 | HQ168670 |
| *Entomacrodus chiostictus* | SIO 07-120-1 | Enci | La Paz, Mexico | HQ168561 | HQ169034 | HQ168789 | HQ168911 | HQ168671 |
| *Entomacrodus nigricans* | KU 139 | Enni | Carrie Bow Cay, Belize | - | HQ169035 | HQ168790 | HQ168912 | HQ168672 |
| *Entomacrodus niuafoouensis* | KU 5534 | Ennu | Saipan, CNMI | HQ168562 | HQ169036 | HQ168791 | HQ168913 | HQ168673 |
| *Entomacrodus striatus* | KU 7061 | Enst | Mahe, Seychelles | HQ168563 | HQ169037 | HQ168792 | HQ168914 | HQ168674 |
| *Hypsoblennius brevipinnis* | SIO 01-41-1 | Hybr | Huatulco, Mexico | HQ168564 | HQ169038 | HQ168793 | HQ168915 | HQ168675 |
| *Hypsoblennius caulopus* | SIO 01-170-1 | Hyca | Golfo de Fonseca, El Salvador | HQ168565 | HQ169039 | HQ168794 | HQ168916 | HQ168676 |
| *Hypsoblennius gentilis* | SIO 06-51-1 | Hyge | Bahia de los Angeles, Mexico | HQ168566 | HQ169040 | HQ168795 | HQ168917 | HQ168677 |
| *Hypsoblennius gilberti* | SIO 05-81-1 | Hygi | Point Loma, California | HQ168567 | - | HQ168796 | HQ168918 | HQ168678 |
| *Hypsoblennius hentzi* | KU 19 | Hyhe | Charleston Harbor, South Carolina, USA | HQ168568 | HQ169041 | HQ168797 | HQ168919 | HQ168679 |
| *Hypsoblennius jenkinsi* | SIO 05-82-1 | Hyje | Point Loma, California, USA | HQ168569 | HQ169042 | HQ168798 | HQ168920 | HQ168680 |
| *Istiblennius dussumieri* | KU 4801 | Isdu | Vanua Levu, Fiji | HQ168570 | HQ169043 | HQ168799 | HQ168921 | HQ168681 |
| *Istiblennius edentulus* | ASIZP 0800861 | Ised | Kenting, Taiwan | HQ168571 | HQ169044 | HQ168800 | HQ168922 | HQ168682 |
| *Istiblennius lineatus* | ASIZP 0800864 | Isli | Kenting, Taiwan | HQ168572 | HQ169045 | HQ168801 | HQ168923 | HQ168683 |
| *Meiacanthus oualanensis* | KU 4133 | Meou | Viti Levu, Fiji | HQ168573 | HQ169046 | HQ168802 | HQ168924 | HQ168684 |
| *Nannosalarias nativitatis* | KU 4041 | Nana | Viti Levu, Fiji | - | - | HQ168803 | HQ168925 | HQ168685 |
| *Omobranchus anolius* | AM 40863-002 | Oman | Black Wattle Bay, Australia | HQ168575 | HQ169048 | HQ168804 | HQ168926 | HQ168686 |
| *Omobranchus obliquus* | KU 4549 | Omob | Viti Levu, Fiji | HQ168576 | - | HQ168805 | HQ168927 | HQ168687 |
| *Ophioblennius macclurei* | KU 136 | Opat | Carrie Bow Cay, Belize | HQ168577 | - | HQ168806 | HQ168928 | HQ168688 |
| *Ophioblennius steindachneri* | SIO 01-43-1 | Opst | Huatulco, Mexico | HQ168578 | HQ169050 | HQ168807 | HQ168929 | HQ168689 |
| *Parablennius marmoreus* | SIO 00-181-1 | Pama | Bocas del Toro, Panama | HQ168579 | - | HQ168808 | HQ168930 | HQ168690 |
| *Petroscirtes mitratus* | KU 7130 | Pemi | Cap Ternay, Seychelles | HQ168580 | HQ169052 | HQ168809 | HQ168931 | HQ168691 |
| *Plagiotremus azaleus* | SIO 01-50-1 | Plaz | Playa Entrega, Mexico | HQ168581 | HQ169053 | HQ168810 | HQ168932 | HQ168692 |
| *Plagiotremus rhinorhnychos* | KU 5019 | Plrh | Scottburgh, South Africa | HQ168582 | HQ169054 | HQ168811 | HQ168933 | HQ168693 |
| *Plagiotremus tapeinosoma* | KU 4501 | Plta | Viti Levu, Fiji | HQ168583 | HQ169055 | HQ168812 | HQ168934 | - |
| *Praealticus caesius* | KU 775 | Prca | Tongatapu, Tonga | - | HQ169056 | HQ168813 | HQ168935 | HQ168694 |
| *Praealticus labrovittatus* | KU 7561 | Prla | Mangilao, Guam | HQ168584 | HQ169057 | HQ168814 | HQ168936 | HQ168695 |
| *Praealticus margaritatus* | ASIZP 0800867 | Prma | Kenting, Taiwan | - | HQ169058 | HQ168815 | HQ168937 | HQ168696 |
| *Praealticus striatus* | ASIZP 0800872 | Prst | Kenting, Taiwan | - | HQ169059 | HQ168816 | HQ168938 | HQ168697 |
| *Praealticus tanegasimae* | ASIZP 0800887 | Prta | Kenting, Taiwan | - | HQ169060 | HQ168817 | HQ168939 | HQ168698 |
| *Rhabdoblennius nitidus* | KU 7544 | Rhel | Yona, Guam | HQ168585 | - | HQ168818 | HQ168940 | HQ168699 |
| *Salarias alboguttatus* | KU 4418 | Saal | Viti Levu, Fiji | HQ168586 | HQ169061 | HQ168819 | HQ168941 | HQ168700 |
| *Salarias fasciatus* | KU 4005 | Safa | Viti Levu, Fiji | HQ168587 | HQ169062 | HQ168820 | HQ168942 | HQ168701 |
| *Xiphasia setifer* | ASIZP 0061269 | Xise | Yilan, Taiwan | HQ168588 | HQ169063 | HQ168821 | - | HQ168702 |
|  |  |  |  |  |  |  |  |  |
| **Family Labrisomidae (36)** |  |  |  |  |  |  |  |  |
| *Alloclinus holderi* | SIO 04-44-1 | Alho | La Jolla, California, USA | HQ168589 | HQ169064 | HQ168822 | HQ168943 | HQ168703 |
| *Auchenionchus microcirrhis* | SIO 03-84-2 | Aumi | Valparaiso, Chile | HQ168590 | HQ169065 | HQ168823 | HQ168944 | HQ168704 |
| *Calliclinus geniguttatus* | SIO 03-84-2 | Cage | Valparaiso, Chile | HQ168591 | HQ169066 | HQ168824 | HQ168945 | HQ168705 |
| *Dialommus macrocephalus* | SIO 03-1-1 | Mnma | Guanacaste, Costa Rica | HQ168592 | HQ169067 | HQ168825 | HQ168946 | HQ168706 |
| *Exerpes asper* | SIO 06-56-1 | Exas | Bahia de los Angeles, Mexico | HQ168593 | HQ169068 | HQ168826 | HQ168947 | HQ168707 |
| *Labrisomus bucciferus* | KU 162 | Labu | Carrie Bow Cay, Belize | HQ168594 | HQ169069 | HQ168827 | HQ168948 | HQ168708 |
| *Labrisomus guppyi* | KU 156 | Lagu | Carrie Bow Cay, Belize | HQ168595 | HQ169070 | HQ168828 | HQ168949 | HQ168709 |
| *Labrisomus haitiensis* | KU 228 | Laha | Carrie Bow Cay, Belize | - | HQ169071 | HQ168829 | HQ168950 | HQ168710 |
| *Labrisomus nigricinctus* | KU 163 | Lani | Carrie Bow Cay, Belize | HQ168596 | HQ169072 | HQ168830 | HQ168951 | HQ168711 |
| *Labrisomus nuchipinnis* | SIO 07-80-1 | Lanu | Los Farallones, Panama | HQ168597 | HQ169073 | HQ168831 | HQ168952 | HQ168712 |
| *Labrisomus striatus* | SIO 07-39-2 | Last | Punta Gordo, Mexico | HQ168598 | HQ169074 | HQ168832 | HQ168953 | HQ168713 |
| *Labrisomus xanti* | SIO 98-34-1 | Laxa | Bahia de los Angeles, Mexico | HQ168599 | HQ169075 | HQ168833 | HQ168954 | HQ168714 |
| *Malacoctenus aurolineatus* | KU 158 | Maau | Carrie Bow Cay, Belize | HQ168600 | HQ169076 | HQ168834 | HQ168955 | HQ168715 |
| *Malacoctenus boehlkei* | KU 182 | Mabo | Carrie Bow Cay, Belize | HQ168601 | HQ169077 | HQ168835 | HQ168956 | HQ168716 |
| *Malacoctenus ebisui* | SIO 01-48-1 | Maeb | Huatulco, Mexico | HQ168602 | HQ169078 | HQ168836 | HQ168957 | HQ168717 |
| *Malacoctenus gigas* | SIO 06-54-1 | Magi | Bahia de los Angeles, Mexico | HQ168603 | HQ169079 | HQ168837 | HQ168958 | HQ168718 |
| *Malacoctenus hubbsi* | SIO 06-54-1 | Mahu | Bahia de los Angeles, Mexico | HQ168604 | HQ169080 | HQ168838 | HQ168959 | HQ168719 |
| *Malacoctenus tetranemus* | SIO 01-40-1 | Mate | Huatulco, Mexico | HQ168605 | HQ169081 | HQ168839 | HQ168960 | HQ168720 |
| *Malacoctenus triangulatus* | SIO 01-127-1 | Matr | Dry Tortugas, Florida, USA | HQ168606 | HQ169082 | HQ168840 | HQ168961 | HQ168721 |
| *Malacoctenus zacae* | SIO 07-2-1 | Maza | Cabo San Lucas, Mexico | HQ168607 | HQ169083 | HQ168841 | HQ168962 | HQ168722 |
| *Malacoctenus zonifer* | SIO 01-170-1 | Mazf | Golfo de Fonseca, El Salvador | HQ168608 | HQ169084 | HQ168842 | HQ168963 | HQ168723 |
| *Malacoctenus zonogaster* | SIO 02-88-1 | Mazo | Isla Floreana, Islas Galapagos | HQ168609 | HQ169085 | HQ168843 | HQ168964 | HQ168724 |
| *Paraclinus integripinnis* | SIO 04-46-1 | Pain | Point Loma, California, USA | HQ168610 | HQ169086 | HQ168844 | HQ168965 | HQ168725 |
| *Paraclinus marmoratus* | KU 183 | Pamr | Carrie Bow Cay, Belize | HQ168611 | HQ169087 | HQ168845 | HQ168966 | HQ168726 |
| *Paraclinus mexicanus* | SIO 01-170-1 | Pame | Golfo de Fonseca, El Salvador | HQ168612 | HQ169088 | HQ168846 | HQ168967 | HQ168727 |
| *Paraclinus sini* | SIO 03-77-1 | Pasi | Loreto, Mexico | HQ168613 | - | HQ168847 | HQ168968 | HQ168728 |
| *Starksia atlantica* | KU 165 | Stat | Carrie Bow Cay, Belize | HQ168614 | HQ169089 | HQ168848 | HQ168969 | HQ168729 |
| *Starksia langi* | KU 164 | Stfa | Carrie Bow Cay, Belize | HQ168615 | HQ169090 | HQ168849 | HQ168970 | HQ168730 |
| *Starksia galapagensis* | SIO 02-89-1 | Stga | Isla Floreana, Islas Galapagos | HQ168616 | HQ169091 | HQ168850 | HQ168971 | HQ168731 |
| *Starksia grammilaga* | SIO 07-124-1 | Stgr | Cabo San Lucas, Mexico | HQ168617 | HQ169092 | HQ168851 | HQ168972 | HQ168732 |
| *Starksia lepicoelia* | KU 226 | Stle | Carrie Bow Cay, Belize | HQ168618 | HQ169093 | HQ168852 | HQ168973 | HQ168733 |
| *Starksia nanodes* | KU 184 | Stna | Carrie Bow Cay, Belize | HQ168619 | - | HQ168853 | HQ168974 | HQ168734 |
| *Starksia occidentalis* | KU 240 | Stoc | Carrie Bow Cay, Belize | HQ168620 | HQ169094 | HQ168854 | HQ168975 | HQ168735 |
| *Starksia posthon* | SIO 01-182-1 | St po | Puerto Vallarta, Mexico | HQ168621 | HQ169095 | - | HQ168976 | HQ168736 |
| *Starksia spinipenis* | SIO 01-182-2 | Stsp | Puerto Vallarta, Mexico | HQ168622 | HQ169096 | HQ168855 | HQ168977 | HQ168737 |
| *Xenomedea rhodopyga* | SIO 04-121 | Xerh | Las Cuevatas, Mexico | - | HQ169097 | HQ168856 | HQ168978 | HQ168738 |
|  |  |  |  |  |  |  |  |  |
| **Family Clinidae (14)** |  |  |  |  |  |  |  |  |
| *Blennioclinus stella* | C24 | Blse | Port Alfred, South Africa | - | HQ169098 | HQ168857 | HQ168979 | HQ168739 |
| *Blennophis striatus* | KU 6477 | Blst | Port Alfred, South Africa | - | HQ169099 | HQ168858 | HQ168980 | HQ168740 |
| *Clinus superciliosus* | KU 6485 | Clsu | Port Alfred, South Africa | JF493223 | HQ169100 | HQ168859 | HQ168981 | HQ168741 |
| *Clinus cottoides* | KU 6487 | Clco | Port Alfred, South Africa | JF493222 | HQ169101 | HQ168860 | HQ168982 | HQ168742 |
| *Cristiceps australis* | C85 | Crau | Port Philip Bay, Australia | HQ168625 | HQ169102 | HQ168861 | HQ168983 | HQ168743 |
| *Gibbonsia elegans* | SIO 02-24-1 | Giel | La Jolla, California | HQ168626 | - | HQ168862 | HQ168984 | HQ168744 |
| *Gibbonsia metzi* | SIO 06-267-1 | Gime | El Rosario, Mexico | HQ168627 | HQ169103 | HQ168863 | HQ168985 | HQ168745 |
| *Gibbonsia montereyensis* | SIO 06-41-1 | Gimo | Vandenburg , California, USA | HQ168628 | - | HQ168864 | HQ168986 | HQ168746 |
| *Heteroclinus adelaidae* | C80 | Head | Flinders Bay, Australia | HQ168629 | HQ169104 | HQ168865 | HQ168987 | HQ168747 |
| *Heteroclinus nasutus* | AM 41084-022 | Hena | Watsons Bay, Australia | HQ168630 | HQ169105 | - | - | - |
| *Heterostichus rostratus* | SIO 01-179-1 | Hero | Mission Bay, California, USA | HQ168631 | HQ169106 | HQ168866 | HQ168988 | HQ168748 |
| *Muraenoclinus dorsalis* | KU 6489 | Mudo | Port Alfred, South Africa | JF493915 | HQ169107 | HQ168867 | HQ168989 | HQ168749 |
| *Pavoclinus graminis* | C23 | Pagr | Port Alfred, South Africa | HQ168633 | HQ169108 | HQ168868 | HQ168990 | HQ168750 |
| *Pavoclinus profundus* | KU 6476 | Papr | Port Alfred, South Africa | HQ168634 | - | HQ168869 | HQ168991 | HQ168751 |
|  |  |  |  |  |  |  |  |  |
| **Family Chaenopsidae (30)** |  |  |  |  |  |  |  |  |
| *Acanthemblemaria aspera* | SIO 01-9-1 | Acas | San Blas, Panama | FJ381429 | FJ381579 | FJ381505 | FJ381543 | FJ381466 |
| *Acanthemblemaria betinensis* | SIO 03-141-1 | Acbe | Bahia Azul, Panama | FJ381430 | FJ381580 | FJ381506 | FJ381544 | FJ381467 |
| *Acanthemblemaria castroi* | SIO 02-89-1 | Acca | Isla Bartolome, Islas Galapagos | FJ381431 | FJ381581 | FJ381507 | FJ381545 | FJ381468 |
| *Acanthemblemaria exilispinus* | SIO 03-142-1 | Acex | Isla Taboga, Panama | FJ381435 | FJ381583 | FJ381511 | FJ381548 | FJ381472 |
| *Acanthemblemaria greenfieldi* | SIO 03-147-1 | Acgr | Carrie Bow Cay, Belize | FJ381436 | FJ381584 | FJ381512 | FJ381549 | FJ381473 |
| *Acanthemblemaria rivasi* | SIO 01-9-1 | Acri | San Blas, Panama | FJ381440 | FJ381588 | FJ381516 | FJ381552 | FJ381477 |
| *Chaenopsis alepidota* | SIO 00-9-1 | Chal | Santa Catalina, California, USA | FJ381442 | FJ381589 | FJ381517 | FJ381553 | FJ381479 |
| *Chaenopsis schmitti* | SIO 02-83-1 | Chsc | Isla Rabida, Islas Galapagos | FJ381444 | FJ381590 | FJ381519 | FJ381555 | FJ381481 |
| *Cirriemblemaria lucasana* | SIO 05-141-1 | Cilu | Bahia Banderas, Mexico | FJ381445 | FJ381591 | FJ381520 | FJ381556 | FJ381482 |
| *Coralliozetus angelicus* | SIO 05-124-1 | Coan | Isla Danzante, Mexico | FJ381446 | FJ381592 | FJ381521 | FJ381557 | FJ381483 |
| *Coralliozetus boehlkei* | SIO 01-52-1 | Cobo | Huatulco, Mexico | FJ381447 | FJ381593 | FJ381522 | FJ381558 | FJ381484 |
| *Coralliozetus cardonae* | SIO 01-5-2 | Coca | Soufrierre, St. Lucia | FJ381448 | FJ381594 | FJ381523 | FJ381559 | FJ381485 |
| *Coralliozetus micropes* | SIO 03-82-2 | Comi | Loreto, Mexico | FJ381449 | FJ381595 | FJ381524 | FJ381560 | FJ381486 |
| *Coralliozetus rosenblatti* | SIO 07-120-1 | Coro | La Paz, Mexico | FJ381429 | FJ381596 | FJ381525 | - | FJ381487 |
| *Coralliozetus springeri* | SIO 01-164-1 | Cosp | Taboquilla, Panama | FJ381429 | FJ381597 | FJ381526 | FJ381561 | FJ381488 |
| *Ekemblemaria myersi* | SIO 01-170-1 | Ekmy | Fonseca, El Savador | FJ381450 | FJ381598 | FJ381527 | FJ381562 | FJ381489 |
| *Ekemblemaria nigra* | SIO 03-141-1 | Ekni | Bahia Azul, Panama | FJ381451 | FJ381599 | - | FJ381563 | FJ381490 |
| *Emblemaria diphyodontis* | SIO 06-276-1 | Emdi | Laguna Grande del Obispo, Venezuela | FJ381452 | FJ381600 | FJ381528 | FJ381564 | FJ381491 |
| *Emblemaria nivipes* | SIO 01-165-1 | Emni | Cocos Island, Costa Rica | FJ381454 | FJ381602 | FJ381530 | FJ381566 | FJ381493 |
| *Emblemaria piratica* | SIO 01-182-1 | Empi | Puerto Vallarta, Mexico | FJ381456 | FJ381604 | FJ381532 | FJ381568 | FJ381495 |
| *Emblemariopsis randalli* | SIO 06-276-1 | Emra | Laguna Grande del Obispo, Venezuela | FJ381458 | FJ381606 | FJ381534 | FJ381570 |  |
| *Emblemariopsis signifer* | SIO 01-171-1 | Emsi | Rio de Janeiro, Brazil | FJ381457 | FJ381605 | FJ381533 | FJ381569 | FJ381496 |
| *Hemiemblemaria simulus* | SIO 05-2-1 | Hesi | Florida Keys, Florida, USA | FJ381459 | FJ381607 | FJ381535 | FJ381571 | FJ381497 |
| *Lucayablennius zingaro* | KU 110 | Luzi | Carrie Bow Cay, Belize | HQ654586 | FJ381608 | FJ381536 | FJ381572 | FJ381498 |
| *Mccoskerichthys sandae* | SIO 01-167-1 | Mcsa | Isla Montuosa, Panama | FJ381461 | FJ381609 | FJ381537 | FJ381573 | FJ381499 |
| *Neoclinus blanchardi* | SIO 00-73-1 | Nebl | Redondo Beach, California, USA | FJ381462 | FJ381610 | FJ381538 | FJ381574 | FJ381500 |
| *Protemblemaria bicirrus* | SIO 02-16-1 | Prbi | La Paz, Mexico | FJ381429 | FJ381611 | FJ381539 | FJ381575 | FJ381501 |
| *Stathmonotus culebrai* | SIO 01-164-1 | Stcu | Taboquilla, Panama | FJ381463 | FJ381612 | FJ381540 | FJ381576 | FJ381502 |
| *Stathmonotus lugubris* | SIO 01-182-1 | Stlu | Puerto Vallarta, Mexico | -- | - | FJ381541 | FJ381577 | FJ381503 |
| *Stathmonotus stahli* | KU 236 | Stst | Carrie Bow Cay, Belize | FJ381464 | - | FJ381542 | FJ381578 | FJ381504 |
|  |  |  |  |  |  |  |  |  |
| **Dactyloscopidae (4)** |  |  |  |  |  |  |  |  |
| *Dactyloscopus lacteus* | SIO 02-88-1 | Dala | Isla Floreana, Islas Galapagos | HQ168635 | HQ169109 | HQ168870 | HQ168992 | HQ168752 |
| *Dactyloscopus pectoralis* | SIO 01-182-1 | Dape | Puerto Vallarta, Mexico | HQ168636 | HQ169110 | - | - | HQ168753 |
| *Gillellus uranidea* | SIO 07-78-1 | Giur | San Blas Islands, Panama | JQ840510 | HQ169112 | HQ168871 | HQ168993 | HQ168754 |
| *Platygillellus rubrocinctus* | KU 206 | Plru | Carrie Bow Cay, Belize | HQ168637 | HQ169113 | HQ168872 | HQ168994 | HQ168755 |
|  |  |  |  |  |  |  |  |  |
| **Family Gobiesocidae (6)** |  |  |  |  |  |  |  |  |
| *Gobiesox juradoensis* | SIO 03-42-1 | Goju | Golfo de San Miguel, Panama | - | - | HQ168873 | HQ168997 | - |
| *Gobiesox pinniger* | SIO 06-51-1 | Gopi | Bahia de los Angeles, Mexico | HQ168638 | - | HQ168874 | HQ168998 | - |
| *Gobiesox rhessodon* | SIO 09-170 | Gorh | La Jolla, California, USA | - | - | HQ168875 | HQ168999 | DQ533377 |
| *Tomicodon humeralis* | SIO 02-1-1 | Tohu | Bahia de los Angeles, Mexico | HQ168639 | - | HQ168876 | HQ169001 | HQ168757 |
| *Tomicodon myersi* | SIO 07-2-1 | Tomy | Cabo San Lucas, Mexico | - | - | HQ168877 | HQ169002 | HQ168758 |
| *Tomicodon zebra* | SIO 07-2-1 | Toze | Cabo San Lucas, Mexico | - | - | HQ168878 | - | HQ168760 |
|  |  |  |  |  |  |  |  |  |
| **Family Grammatidae (1)** |  |  |  |  |  |  |  |  |
| *Gramma loreto* | GenBank | Grlo | Genbank | AY662751 | AY539461 | EU167836 | - | AY539262 |
|  |  |  |  |  |  |  |  |  |
| **Family Opistognathidae (1)** |  |  |  |  |  |  |  |  |
| *Opistognathus aurifrons* | GenBank | Opau | Genbank | FJ583762 | JX560320 | EU167852 | - | DQ533420 |
|  |  |  |  |  |  |  |  |  |
