# Supplementary figures and images for: Phylogeny and biogeography of a shallow water fish clade (Teleostei: Blenniiformes)
Source: BMC Evol Biol. 2013 Sep 25;13:210. doi: 10.1186/1471-2148-13-210 (PMC3849733; doi:10.1186/1471-2148-13-210)

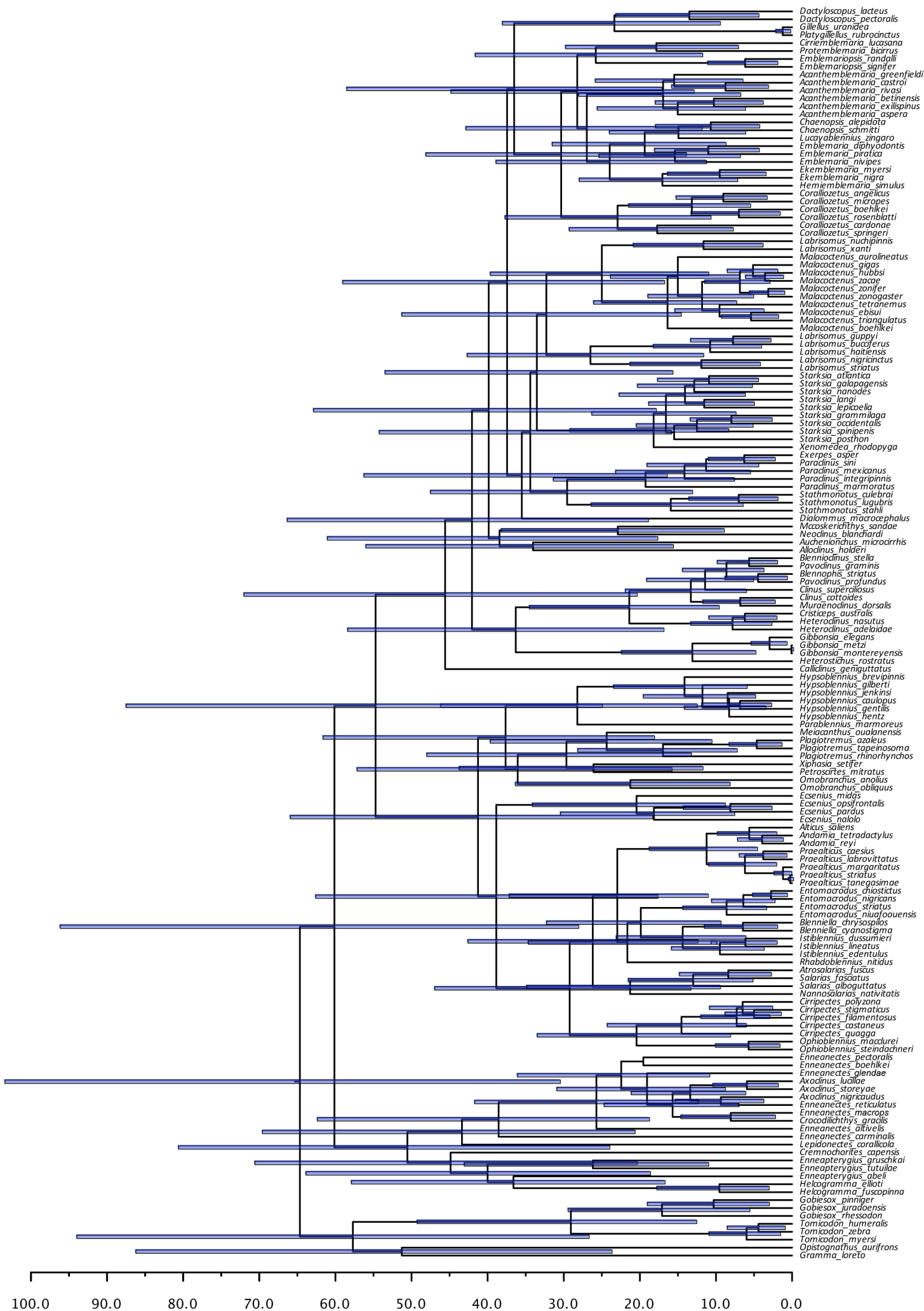

Supplement: Additional file 2: Figure S1 — Posterior maximum clade credibility relative time tree of blenniiform species inferred from a relaxed molecular clock analysis using BEAST. Branches are scaled to age estimates. Bars at nodes reflect the 95% highest posterior density of the age estimates. [file 1471-2148-13-210-S2.pdf]

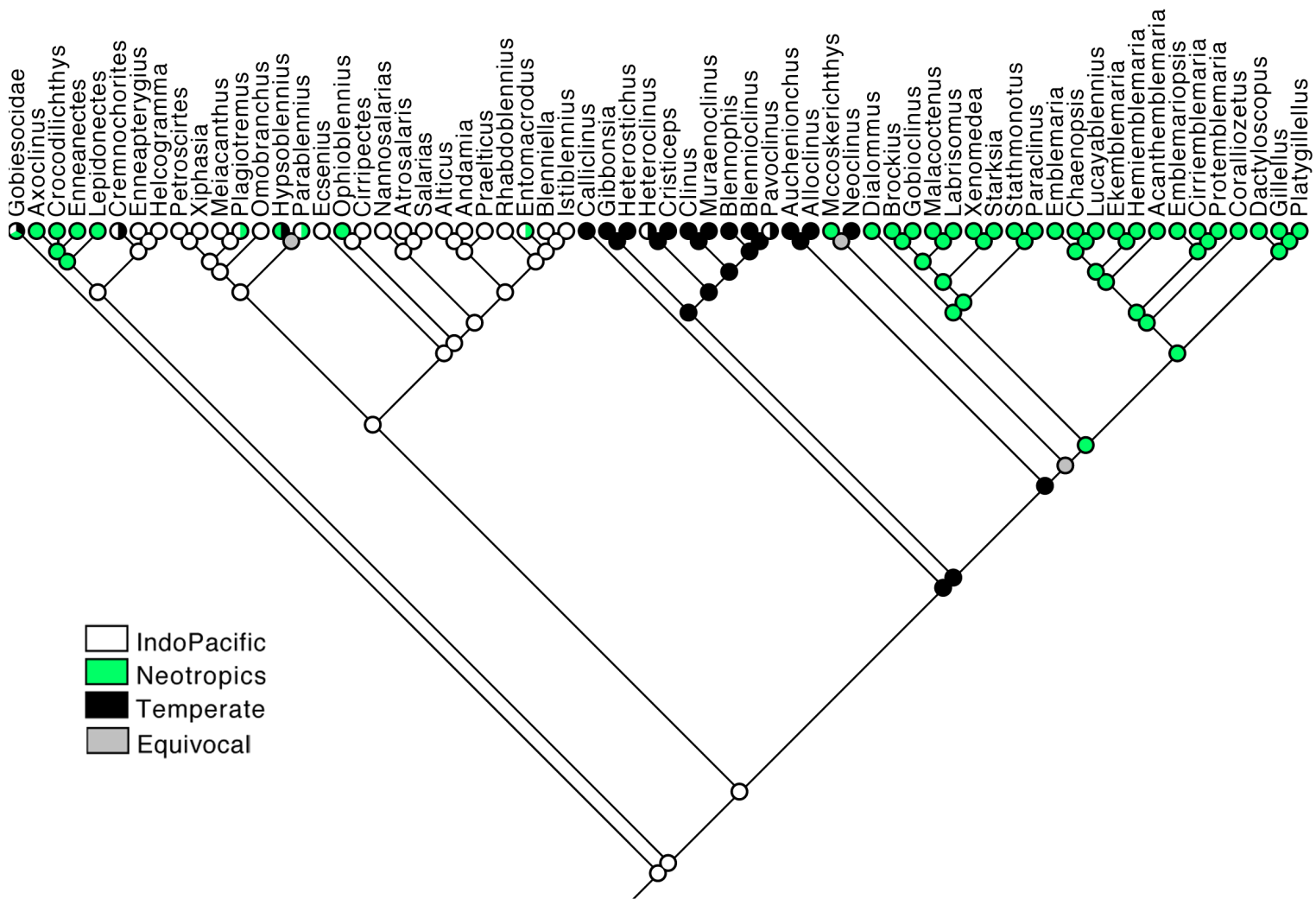

Supplement: Additional file 3: Figure S2 — Ancestral distribution at each node of the blenniiform phylogeny estimated by MP analysis implemented in Mesquite. [file 1471-2148-13-210-S3.pdf]
