# Supplementary material for: Phylogeny and biogeography of a shallow water fish clade (Teleostei: Blenniiformes)
Source: BMC Evol Biol. 2013 Sep 25;13:210. doi: 10.1186/1471-2148-13-210 (PMC3849733; doi:10.1186/1471-2148-13-210)
Supplement: Additional file 4: Table S2 — Biogeographical distribution of blenniiform genera. [file 1471-2148-13-210-S4.docx]

Table S2. Biogeographical distribution of blenniiform genera sampled in this study. I: tropical Indo-Pacific; T: temperate waters; N: Neotropics.

| Genus | Distribution |
| --- | --- |
| *Acanthemblemaria* | N |
| *Alloclinus* | T |
| *Alticus* | I |
| *Andamia* | I |
| *Atrosalaris* | I |
| *Auchenionchus* | T |
| *Axoclinus* | N |
| *Blenniella* | I |
| *Blennioclinus* | T |
| *Blennophis* | T |
| *Brockius* | N |
| *Calliclinus* | T |
| *Chaenopsis* | N |
| *Cirriemblemaria* | N |
| *Cirripectes* | I |
| *Clinus* | T |
| *Coralliozetus* | N |
| *Cremnochorites* | IT |
| *Cristiceps* | T |
| *Crocodilichthys* | N |
| *Dactyloscopus* | N |
| *Dialommus* | N |
| *Ecsenius* | I |
| *Ekemblemaria* | N |
| *Emblemaria* | N |
| *Emblemariopsis* | N |
| *Enneanectes* | N |
| *Enneapterygius* | I |
| *Entomacrodus* | IN |
| *Gibbonsia* | T |
| *Gillellus* | N |
| *Gobioclinus* | N |
| *Helcogramma* | I |
| *Hemiemblemaria* | N |
| *Heteroclinus* | IT |
| *Heterostichus* | T |
| *Hypsoblennius* | TN |
| *Istiblennius* | I |
| *Labrisomus* | N |
| *Lepidonectes* | N |
| *Lucayablennius* | N |
| *Malacoctenus* | N |
| *Mccoskerichthys* | N |
| *Meiacanthus* | I |
| *Muraenoclinus* | T |
| *Nannosalarias* | I |
| *Neoclinus* | T |
| *Omobranchus* | I |
| *Ophioblennius* | N |
| *Parablennius* | ITN |
| *Paraclinus* | N |
| *Pavoclinus* | IT |
| *Petroscirtes* | I |
| *Plagiotremus* | IN |
| *Platygillellus* | N |
| *Praelticus* | I |
| *Protemblemaria* | N |
| *Rhabdoblennius* | I |
| *Salarias* | I |
| *Starksia* | N |
| *Stathmonotus* | N |
| *Xenomedea* | N |
| *Xiphasia* | I |
| *Gobiesocidae* | ITN |
